# Terpenoids from the Deep-Sea-Derived Fungus *Penicillium* *thomii* YPGA3 and Their Bioactivities

**DOI:** 10.3390/md18030164

**Published:** 2020-03-16

**Authors:** Zhongbin Cheng, Wan Liu, Runzhu Fan, Shouye Han, Yuanli Li, Xiaoyun Cui, Jia Zhang, Yinan Wu, Xin Lv, Yun Zhang, Zhuhua Luo, Siti Aisyah Alias, Wei Xu, Qin Li

**Affiliations:** 1 School of Pharmacy, Henan University, Kaifeng 475004, China; 18737806806@163.com (W.L.); hanshouye123@163.com (S.H.);; 2 Key Laboratory of Marine Biogenetic Resources, Third Institute of Oceanography, Ministry of Natural Resources, Xiamen 361005, China; luozhuhua@tio.org.cn; 3 School of Pharmaceutical Sciences, Sun Yat-sen University, Guangzhou 510006, China; 4 Eucommia Ulmoides Cultivation and Utilization of Henan Engineering Laboratory, Kaifeng 475004, China; 5 Institute of Ocean and Earth Science (IOES), C308, Institute of Postgraduate Studies Building, University of Malaya, Kuala Lumpur 50603, Malaysia

**Keywords:** *Penicillium thomii* YPGA3, deep-sea-derived fungus, austalide meroterpenoid, labdane-type diterpenoid, bioactivities

## Abstract

A chemical study of the ethyl acetate (EtOAc) extract from the deep-sea-derived fungus *Penicillium* *thomii* YPGA3 led to the isolation of a new austalide meroterpenoid (**1**) and seven known analogues (**2**−**8**), two new labdane-type diterpenoids (**9** and **10**) and a known derivative (**11**). The structures of new compounds **1**, **9**, and **10** were determined by comprehensive analyses via nuclear magnetic resonance (NMR) and mass spectroscopy (MS) data. The absolute configurations of **1**, **9**, and **10** were determined by comparisons of experimental electronic circular dichroism (ECD) with the calculated ECD spectra. Compound **1** represented the third example of austalides bearing a hydroxyl group at C-5 instead of the conserved methoxy in other known analogues. To our knowledge, diterpenoids belonging to the labdane-type were discovered from species of *Penicillium* for the first time. Compound **1** showed cytotoxicity toward MDA-MB-468 cells with an IC_50_ value of 38.9 μM. Compounds **2** and **11** exhibited inhibition against α-glucosidase with IC_50_ values of 910 and 525 μM, respectively, being more active than the positive control acarbose (1.33 mM).

## 1. Introduction

Austalides are a class of natural meroterpenoids with attractive scaffolds. Previous biosynthetic studies revealed that they are biosynthesized by cyclization and oxidative modification of 6-[(2*E*, 6*E*)farnesyl]-5,7-dihydroxy-4-methylphthalide [1]. These meroterpenoids were mainly produced by the species of the fungal genera *Aspergillus* and *Penicillium*, especially those from marine environments. Since austalides A−E were first reported in 1981, a total of 36 analogues have been identified [2,3,4,5,6,7,8,9,10,11]. The structural variations of austalides are attributed to oxidation occurring at C-13, C-14, C-17, and the isopropyl (C-15, C-25, C-26) to generate alcohol, isopropenyl, lactone, ester, or ether functionalities. Some of the analogues exhibited significant bioactivities, such as cytotoxicity [6,10], antibacterial activity [7], anti-influenza A virus (H1N1) activity [8], endo-1,3-*D*-glucanase inhibition [9], and osteoclast differentiation inhibitory effects [11]. 

**Figure 1:**
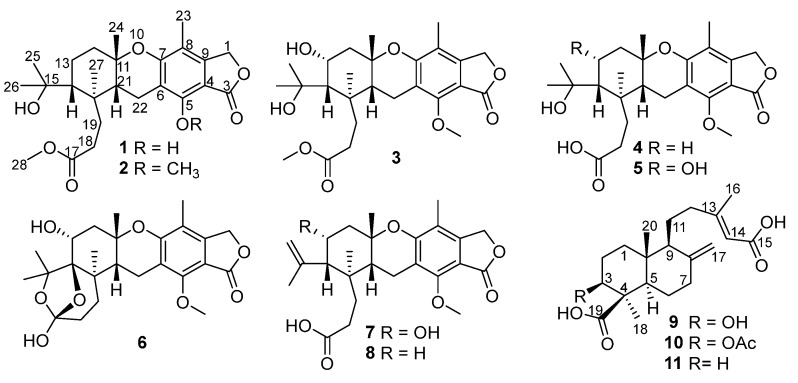
Structures of compounds **1**–**11** from *Penicillium thomii* YPGA3.

In our efforts to search for new or bioactive molecules from deep-sea-derived *Penicillium* or *Aspergillus* strains [12,13,14,15], chemical examination of *Penicillium thomii* YPGA3 afforded a new austalide meroterpenoid (**1**) and seven known analogues (**2**−**8**) (Figure 1). Compound **1** represented the third example of austalides bearing a phenol hydroxy at C-5 instead of the conserved methoxy in other known analogues. Additionally, two new labdane-type diterpenoids (**9** and **10**) and a known analogue (**11**) were also obtained (Figure 1). The labdane-type diterpenoids are mainly produced by plants and are rarely found in fungus. To our knowledge, diterpenoids belonging to the labdane-type were discovered from species of *Penicillium* for the first time. All compounds were evaluated for their inhibitions against α-glucosidase and NO (nitric oxide) production in lipopolysaccharide (LPS)-activated RAW 264.7 macrophages, and cytotoxicity toward two types of human breast carcinoma cells. Herein, the isolation, structural elucidation, and the bioactivity of compounds **1**–**11** are described.

## 2. Results

**Figure 2:**
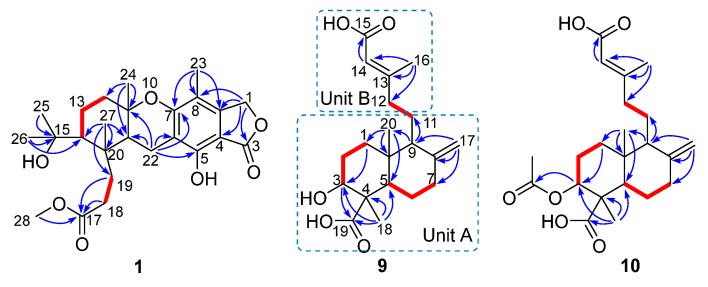
Key correlation spectroscopy (COSY, 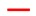) and heteronuclear multiple-bond correlations (HMBC, 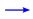) of compounds **1**, **9**, and **10**.

**Figure 3:**
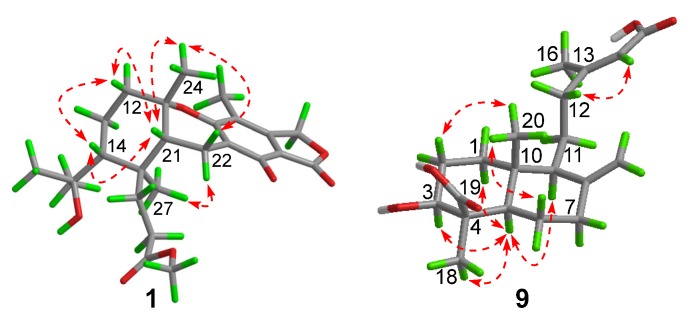
Key nuclear Overhauser effect spectroscopy (NOESY) correlations (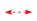) of compounds **1** and **9**.

**Figure 4:**
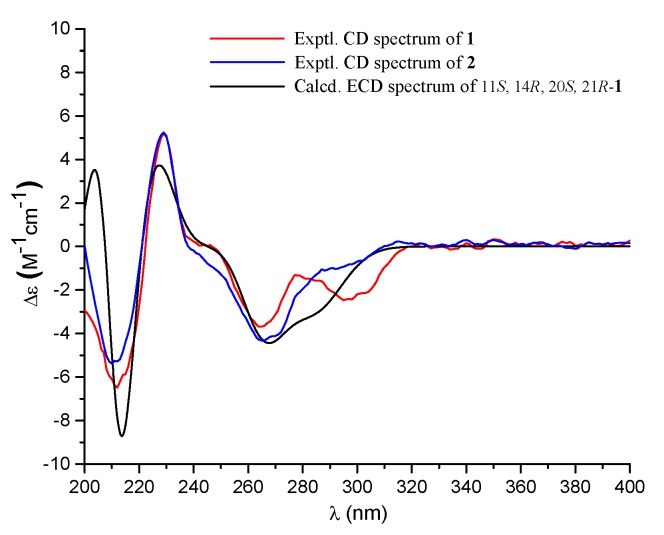
Experimental electronic circular dichroism (ECD) spectra (200–400 nm) of compounds **1** and **2** in methanol and the calculated ECD spectrum of 11*S*, 14*R*, 20*S*, 21*R*-**1** at the B3LYP/6-31+G(d,p) level in methanol.

Compound **1** had a molecular formula of C_25_H_34_O_7_, as established by the high-resolution electrospray ionization mass spectroscopy (HRESIMS) (469.2202 [M + Na]^+^, calcd. for 469.2197) (see Figure S34 in the Supplementary Materials), requiring nine degrees of unsaturation. The ^1^H nuclear magnetic resonance (NMR) spectrum exhibited signals for five methyl singlets (δ_H_ 2.02, 1.28, 1.21 × 2, 0.71), a methoxy (δ_H_ 3.67), an oxygenated methylene (δ_H_ 5.22), and a series of alkyl protons (see Figure S1). The ^13^C NMR and the heteronuclear single-quantum coherence (HSQC) spectra exhibited 25 carbon resonances attributable to a benzene ring (δc 160.8, 153.8, 145.4, 112.3, 103.0, 111.2), five methyls (δc 33.2, 28.2, 27.7, 19.4, 10.6), a methoxy (δc 52.0), six methylenes (δc 70.6, 40.5, 22.6, 30.1, 34.9, 18.4) including an oxygenated one, two methines (δc 41.5, 52.1), two ester carbons (δc 174.2, 177.0), one sp^3^ quaternary carbon (δc 42.8), and two oxygenated carbons (δc 78.2, 75.8) (see Figures S2 and S3). As six degrees of unsaturation were accounted for by the benzene ring and two carbonyl carbons, the remaining three degrees of unsaturation required that compound **1** contained three additional rings. The aforementioned information was very similar to that of austalide P (**2**) [6], a co-isolated analogue first isolated from a sponge-associated fungus *Aspergillus* sp., with the only difference owing to the absence of the aromatic methoxy group (δ_H_ 4.04, δ_C_ 62.2) in **2**. This indicated that **1** was the 5-demethoxylated derivative of **2**. The structure of **1** was further certified as correct by detailed interpretation of 2D NMR data (Figure 2). The relative configuration of **1** was assigned by a nuclear Overhauser effect spectroscopy (NOESY) experiment (Figure 3). The NOE correlations of H-21 (δ_H_ 1.68)/H_3_-24 (δ_H_ 1.21), H-14 (δ_H_ 1.55), H_3_-24/H-22β (δ_H_ 2.69), and H_3_-27 (δ_H_ 0.71)/H-22α (δ_H_ 2.94) clarified the same orientation of H_3_-24, H-21, and H-14, while H_3_-27 was in the opposite orientation. Thus, the relative configuration of **1** was assigned as 11*S*^∗^, 14*R*^∗^, 20*S*^∗^, and 21*R*^∗^. In order to resolve its absolute configuration, the theoretical electronic circular dichroism (ECD) data of 11*S*, 14*R*, 20*S*, 21*R*-**1** were calculated by the time-dependent density-functional theory (TDDFT) method and showed an ECD curve with Cotton effects at 265 (−), 227 (+), and 214 (−) nm, which were in good agreement with the experimental cotton effects at 264 (−), 229 (+), and 212 (−) nm (Figure 4), suggesting that compound **1** has the 11*S*, 14*R*, 20*S*, 21*R* configuration. Compound **1** was given the trivial name austalide Y and represented the third example of an austalide meroterpenoid without the 5-methoxy group. 

**Figure 5:**
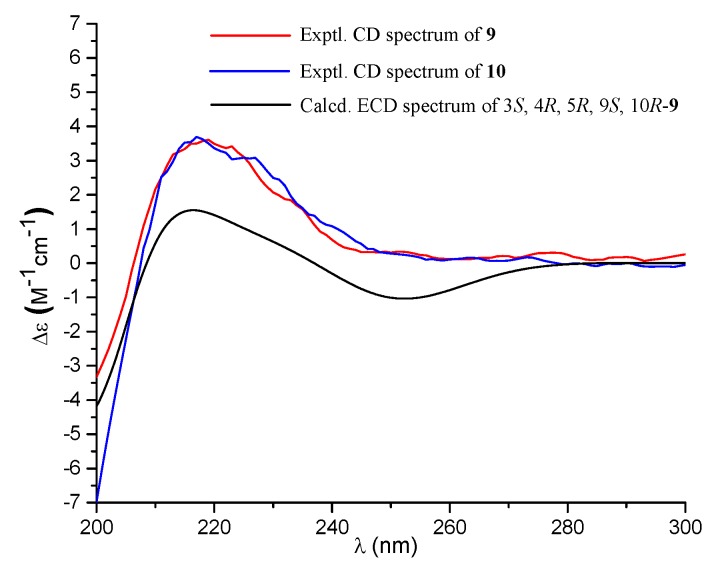
Experimental ECD spectra (200–300 nm) of **9** and **10** in methanol and the calculated ECD spectrum of 3*S*, 4*R*, 5*R*, 9*S*, 10*R*-**9** at the B3LYP/6-31+G(d,p) level in methanol.

Compound **9** was isolated as a colorless oil with the molecular formula of C_20_H_30_O_5_ as determined by the HRESIMS at *m*/*z* 373.1991 [M + Na]^+^ (calcd. 373.1985), requiring six degrees of unsaturation. The ^1^H NMR spectrum exhibited signals for an olefinic methyl (δ_H_ 2.13), two methyl singlets (δ_H_ 1.38 and 0.69), an olefinic methylene (δ_H_ 4.56, 4.90), an oxygenated proton (δ_H_ 3.18), and several aliphatic protons. The ^13^C NMR spectra exhibited 20 carbon resonances, which were classified by an HSQC experiment as three methyls (δ_C_ 24.8, 18.9, 13.5), six sp^3^ methylenes (δ_C_ 40.7, 39.6, 38.7, 29.7, 27.2, 23.0), three methines (δ_C_ 79.0, 56.5 × 2) including one oxygenated methine, two sp^3^ quaternary carbons (δ_C_ 50.3, 41.0), two carboxylic acid groups (δ_C_ 170.4, 180.5), and two double bonds (δ_C_ 161.7, 149.0, 107.3, 116.9). As four of the six degrees of unsaturation were covered by two carboxylic acid groups and two double bonds, the remaining two degrees of unsaturation required that **9** was bicyclic. The gross structure was further established by detailed analyses of the 2D NMR data (Figure 2). The correlation spectroscopy (COSY) relationships from H_2_-1 to H-3 and H-5 to H_2_-7 coupled with the heteronuclear multiple-bond correlations (HMBCs) of H_3_-20 (δ_H_ 0.69) to C-1 (δ_C_ 38.7), C-5 (δ_C_ 56.5), C-10 (δ_C_ 41.0), H_3_-18 (δ_H_ 1.38) to C-3 (δ_C_ 79.0), C-4 (δ_C_ 50.3), C-5, C-19 (δ_C_ 180.5), the olefinic methylene protons H_2_-17 (δ_H_ 4.56, 4.90) to C-7 (δ_C_ 39.6), C-8 (δ_C_ 149.0), C-9 (δ_C_ 56.5), and H-9 (δ_H_ 1.61) to C-11 (δ_C_ 23.0), C-20 (δ_C_ 13.5) established a bicyclic unit (unit A). Additional HMBCs from H_3_-16 (δ_H_ 2.13) to C-12 (δ_C_ 40.7), C-13 (δ_C_ 161.7), C-14 (δ_C_ 116.9), and H-14 (δ_H_ 5.61) to C-15 (δ_H_ 170.4) established a senecioic acid moiety (unit B), which was linked to unit A at C-11 by the ^1^H-^1^H COSY relationship between H_2_-11 and H_2_-12. Thus, the gross structure of **9** was established as depicted. The relative configuration of **9** was determined by an NOESY experiment (Figure 3) and coupling constants. The coupling constants of *J*_H-3/H-2__α_ (4.4 Hz) and *J*_H-3/H-2__β_ (12.1 Hz) suggested that OH-3 was *β*-orientated. The NOE correlations of H-5 with H-3, H-9 (δ_H_ 1.61), H_3_-18 (δ_H_ 1.38), H_3_-18 with H-6α (δ_H_ 2.03), and H_3_-20 (δ_H_ 0.69) with H-6β (δ_H_ 1.93) indicated that H-3, H-5, and H-9 were α-orientated, while H_3_-20 was *β*-orientated. The NOE correlation between H-14 and H_2_-12 (δ_H_ 2.30, 2.02) was indicative of an *E* configuration of the double bond Δ^13^. Comparison of the experimental ECD data with those of the calculated ECD data at the B3LYP/6-31+G(d,p) level for 3*S*, 4*R*, 5*R*, 9*S,* 10*R*-**9** allowed the assignment of the 3*S*, 4*R*, 5*R*, 9*S,* 10*R* configuration for **9** (Figure 5). As the structure of **9** was 3-hydroxylated derivative of agathic acid (**10**) [16], it was named 3*β*-hydroxy-agathic acid. 

Compound **10** had a molecular formula of C_22_H_32_O_6_, as determined by the HRESIMS at *m*/*z* 415.2096 [M + H]^+^ (calcd. 415.2091), requiring seven degrees of unsaturation. The NMR data of **10** were similar to those of **9** with the obvious distinction due to the presence of an acetyl group (δ_H_ 2.04_,_ δ_C_ 172.8), suggesting that **10** was an acetylated derivative of **9**. The downfield-shifted H-3 (δ_H_ 4.55) showed an HMBC with the acetyl carbonyl carbon (δ_C_ 172.8), locating the acetyl group at C-3 (Figure 2). The relative configuration of **10** was determined to be the same as that of **9** based on their similar NOESY data. The structure of **10** was determined as depicted and is a C-3 epimer of a known analogue mumic acid A [17]. The similar specific rotations and circular dichroism (CD) spectra of **9** and **10** confirmed the same absolute configuration of both **9** and **10** (Figure 5), and compound **10** was named 3*β*-acetoxy-agathic acid.

In addition, eight additional known compounds were identical to austalide P (**2**) [6], austalide H (**3**) [5], austalide P acid (**4**) [9], austalide H acid (**5**) [9], 17-O-demethylaustalide B (**6**) [9], austalide Q acid (**7)** [9], 13-deoxyaustalide Q acid (**8)** [9], and agathic acid (**10**) [16] based on comparisons of their NMR data and specific rotations with those reported in the literature. Furthermore, the ^13^C NMR data of austalide H (**3**) and ^13^C NMR data of compounds **3**, **4**, and **7** in methanol-*d*_6_ were reported for the first time.

All compounds were screened for their inhibitory activities against *α*-glucosidase at the initial concentration of 1 mM. Compounds **2** and **11** exhibited inhibition by more than 50% and were further evaluated to calculate the IC_50_ values. The results showed that compounds **2** and **11** inhibited *α*-glucosidase with IC_50_ values of 910 ± 4 and 525 ± 2 μM, being more active than the positive control acarbose (1.33 mM). Other compounds, on the other hand, showed inhibition less than 40% at the concentration of 1 mM. As for the labdane-type diterpenoids **9**−**11**, the introduction of hydroxy and acetoxy groups at C-3 may lead to a sharp decrease in activity, since compounds **9** and **10** showed low inhibition when compared with that of **11**.

The isolated compounds were also evaluated for their inhibitory effects against NO production in LPS-activated RAW 264.7 macrophages at the concentration of 50 μM following the same procedures in our previous study [10]. The cell viability was further determined by the MTT assay to evaluate whether the inhibition on NO production was owing to the cytotoxicity. As results (see Table S1 in the Supplementary Materials), compounds **1**, **2**, and **10,** possessing inhibition rates of more than 50% on NO production, showed obvious cytotoxic effects, which suggested that the inhibitory effects of NO production were due to the cytotoxicity. All compounds were further evaluated for their cytotoxicity toward two types of human breast carcinoma cells (MCF-7, MDA-MB-468) [13], and the results showed that only compound **1** showed a weak inhibitory effect toward MDA-MB-468 cells with an IC_50_ value of 38.9 ± 1.83 μM. 

## 3. Experimental Section

### 3.1. General Experimental Procedure 

Specific rotations were recorded by an SGW^®^-1 automatic polarimeter (Shanghai Jing Ke Industrial Co., Ltd., Shanghai, China). The NMR spectra were measured on a Bruker Avance III HD-400 spectrometer (Bruker, Fällanden, Switzerland). HRESIMS spectra were obtained on a Waters Xevo G2 Q-TOF spectrometer (Waters Corporation, Milford, MA, USA). Semi-preparative high-performance liquid chromatography (HPLC) was undertaken on a Shimadzu LC-6AD pump (Shimadzu Co., Kyoto, Japan) using a UV detector, and a YMC-Pack ODS-A HPLC column (semipreparative, 250 × 10 mm, S-5 μm, 12 nm, YMC Co., Ltd, Kyoto, Japan) was used for separation. 

### 3.2. Fungal Strain and Identification 

Fungus YPGA3 was isolated from deep sea water at a depth of 4500 m in the Yap Trench (West Pacific Ocean). The strain was identified as *Penicillium thomii* based on microscopic examination and by internal transcribed spacer (ITS) sequencing. The ITS sequence was deposited in GenBank (http://www.ncbi.nlm.nih.gov) with accession number MG835903. The strain YPGA3 (MCCC 3A01052) was deposited at the Marine Culture Collection of China.

### 3.3. Fermentation 

The fermentation was carried out in 30 Fernbach flasks (500 mL), each containing 70 g of rice. Artificial seawater (90 mL) was added to each flask, and the contents were soaked for three hours before autoclaving at 15 psi for 30 min. After cooling to room temperature, each flask was inoculated with 3.0 mL of the spore inoculum and incubated at room temperature for 30 days.

### 3.4. Extraction and Isolation 

The fermented materials were extracted with ethyl acetate (EtOAc) (3 × 5000 mL) in an ultrasonic bath at 30 °C for 20 min. After evaporation under vacuum, the EtOAc extract (3.1g) was subjected to ODS silica gel column chromatography (CC) eluting with MeOH/H_2_O (20:80→100:0) to afford ten fractions (F1–F10). F5 was further chromatographed over C-18 silica gel CC eluted with MeOH/H_2_O (65:35) to afford seven subfractions (F5a–F5g). F5d was further purified by HPLC on a semi-preparative YMC-pack ODS-A column using CH_3_CN/H_2_O (61:39, 3 mL/min) to afford **5** (31 mg, t*_R_* 40 min). F6 was further chromatographed over ODS silica gel CC eluted with MeOH/H_2_O (20:80→100:0) to afford fourteen subfractions (F6a–F6n). F6d was separated by HPLC CH_3_CN/H_2_O (54:46, 3 mL/min) to yield **3** (14 mg, t*_R_* 19.9 min). Purification of F6e by HPLC using CH_3_CN/H_2_O (54:46, 3 mL/min) gave **7** (10 mg, t*_R_* 21.9 min). F6h was purified by HPLC using CH_3_CN/H_2_O (52:48, 3 mL/min) to give **9** (3 mg, t*_R_* 23.9 min). F6i was purified by HPLC using CH_3_CN/H_2_O (53:47, 3 mL/min) to afford **4** (5 mg, t*_R_* 49.6 min) and **10** (2 mg, t*_R_* 53.6 min). F6j was purified by HPLC using CH_3_CN/H_2_O (50:50, 3 mL/min) to afford **6** (2 mg, t*_R_* 22.5 min). F6k was purified by HPLC using CH_3_CN/H_2_O (65:35, 3 mL/min) to afford **1** (1 mg, t*_R_* 36.2 min) and **2** (6 mg, t*_R_* 39.0 min). F6l was purified by HPLC using CH_3_CN/H_2_O (68:32, 3 mL/min) to afford **11** (20 mg, t*_R_* 24.4 min). F6m was purified by HPLC using CH_3_CN/H_2_O (70:30, 3 mL/min) to afford **8** (1.5 mg, t*_R_* 48.9 min).

Austalide Y (**1**): colorless oil; ${\left[\alpha \right]}_{D}^{25}$ −33 (*c* 0.05, MeOH); UV (MeOH) *λ*_max_ 222, 270 nm; ECD (*c* 2.24 × 10^−4^ M, MeOH) *λ*_max_ (Δε) 264 (−3.69), 229 (+5.21), 212 (−6.49); ^1^H and ^13^C NMR data, see Table 1; HRESIMS *m*/*z* 469.2202 [M + Na]^+^ (calcd. for C_25_H_34_O_7_Na^+^, 469.2197).

3*β*-Hydroxy-agathic acid (**9**): colorless oil; ${\left[\alpha \right]}_{D}^{25}$ +66 (*c* 0.06, MeOH); ECD (*c* 5.7 × 10^−4^ M, MeOH) *λ*_max_ 217 (4.05); ^1^H and ^13^C NMR data, see Table 2; HRESIMS *m*/*z* 373.1991 [M + Na]^+^ (calcd. for C_20_H_30_O_5_Na^+^, 373.1985).

3*β*-Acetoxy-agathic acid (**10**): colorless oil; ${\left[\alpha \right]}_{D}^{25}$ +58 (*c* 0.03, MeOH); ECD (*c* 5.1 × 10^−4^ M, MeOH) *λ*_max_ 225 (3.78); ^1^H and ^13^C NMR data, see Table 2; HRESIMS *m*/*z* 415.2096 [M + Na]^+^ (calcd. for C_22_H_32_O_6_Na^+^, 415.2091).

### 3.5. α-Glucosidase Assay

The α-glucosidase inhibitory effect was assessed as follows. First, 0.2 U of α-glucosidase from *Saccharomyces cerevisiae* was purchased from Sigma-Aldrich (St. Louis, MO, USA), and was diluted in a 0.067 M phosphate buffer consisting of Na_2_HPO_4_·12H_2_O and KH_2_PO_4_ (pH 6.8). The assay was conducted in a 60 μL reaction system containing 20 μL of diluted enzyme solution, and 20μL of dimethyl sulfoxide (DMSO) or sample (dissolved in DMSO). After 10 min of incubation in 96-well plates at 37 °C, a 20 μL portion of 4 mM 4-nitrophenyl-*α*-d-glucopyranoside (PNPG) (Aladdin, Shanghai, China) was added as a substrate to start the enzymatic reaction. The plate was incubated for an additional 20 min at 37 °C, and the reaction was quenched by adding 60 μL of 0.2 M Na_2_CO_3_. The final concentrations of tested compounds were between 0.2 and 2 mM. The optical density (OD) was measured at an absorbance wavelength of 405 nm using a Microplate Reader (Tecan, Mannedorf, Switzerland). All assays were performed in three replicates, and acarbose (Aladdin, Shanghai, China) was used as the positive control.

## 4. Conclusions

In conclusion, a new austalide meroterpenoid (**1**) and seven known austalide analogues (**2**−**8**), two new labdane-type diterpenes (**9** and **10**) and one known derivative (**11**) were isolated from a fraction of the EtOAc extract of the deep-sea derived strain *Penicillium thomii* YPGA3. The structures of compounds **1**, **9**, and **10** were determined by comprehensive analyses of the NMR and mass spectroscopy (MS) data, the absolute configurations of **1** and **9** were determined by ECD calculations. Compound **1** showed weak inhibition toward MDA-MB-468 cells with an IC_50_ value of 38.9 μM. Compounds **2** and **11** exhibited inhibitory effects against α-glucosidase with IC_50_ values of 910 and 525 μM, respectively, being more active than the positive control acarbose (1.33 mM). 

## Figures and Tables

**Table 1:** Nuclear magnetic resonance (NMR) data of **1**, **3**, **4**, and **7** in Methanol-*d*_4_^a^ (δ in ppm).

		1	3	4	7
No.	δ_H_ (mult., *J* in Hz)	δ_C_	δ_C_	δ_C_	δ_C_
1, CH_2_	5.22, s	70.6	69.8	69.8	69.8
3, C		174.2	171.9	171.9	171.8
4, C		103.0	108.5	108.2	108.7
5, C		160.8	156.6	156.6	156.7
6, C		111.2	117.5	117.4	117.3
7, C		153.8	159.7	160.5	159.5
8, C		112.3	116.6	115.8	116.1
9, C		145.4	147.5	147.5	147.0
11, C		78.2	78.4	78.3	79.2
12, CH_2_	2.12, m 1.63, m	40.5	45.9	40.5	45.9
13, CH_2_/CH	1.84, m 1.52, m	22.6	70.1	22.6	72.2
14, CH	1.55, m	52.1	51.7	51.9	53.4
15, C		75.8	78.4	75.8	147.5,
17, C		177.0	178.6	178.9	177.9
18, CH_2_	2.32, m 2.61, m	30.1	30.5	30.3	30.0
19, CH_2_	2.40, m 1.82, m	34.9	36.2	35.0	36.2
20, C		42.8	42.1	42.8	40.7
21, CH	1.68, d (8.2)	41.5	41.5	41.4	40.7
22, CH_2_	2.69, dd (18.3, 8.2) 2.94, d (18.3)	18.4	18.7	18.8	18.9
23, CH_3_	2.02, s	10.6	10.8	10.6	10.8
24, CH_3_	1.21, s	27.7	28.2	27.8	27.9
25, CH_3_/CH_2_	1.28, s	33.2	32.1	33.2	116.1
26, CH_3_	1.21, s	28.2	32.7	28.2	26.5
27, CH_3_	0.71, s	19.4	18.5	19.6	20.9
COOCH_3_	3.67, s	52.0	52.1		
5-OCH_3_			62.3	62.2	62.3

*^a^*^1^H NMR recorded at 400 MHz, ^13^C NMR recorded at 100 MHz.

**Table 2:** ^1^H and ^13^C NMR Data of **9** and **10** in Methanol-*d*_4_ *^a^* (δ in ppm).

No.	9		10	
	δ_H_ (mult., *J* in Hz)	δ_C_	δ_H_ (mult., *J* in Hz)	δ_C_
1, CH_2_	1.24, m 1.91, m	38.7	1.31, m 1.93, m	38.1
2, CH_2_	1.78, m 2.16, m	29.7	1.72, m 2.46, m	25.7
3, CH	3.18, dd (12.1, 4.4)	79.0	4.55, dd (12.3, 4.5)	80.9
4, C		50.3		49.3
5, CH	1.29, m	56.5	1.43, dd (12.8, 2.4)	56.6
6, CH_2_	1.93, m 2.03, m	27.2	1.63, m 2.04, m	26.9
7, CH_2_	1.93, m 2.42, m	39.6	1.99, m 2.43, m	39.3
8, C		149.0		148.7
9, CH	1.61, m	56.5	1.65, m	56.4
10, C		41.0		40.8
11, CH_2_	1.73, m 1.58, m	23.0	1.75, m 1.58, m	23.0
12, CH_2_	2.02, m 2.30, m	40.7	2.03, m 2.30, m	40.6
13, C		161.7		161.2
14, CH	5.61, s	116.9	5.62, s	115.8
15, C		170.4		170.7
16, CH_3_	2.13, d (1.0)	18.9	2.13, s	18.9
17, CH_2_	4.56, br s 4.90, br s	107.3	4.57, br s 4.92, br s	107.6
18, CH_3_	1.38, s	24.8	1.24, s	24.7
19, C		180.5		177.5
20, CH_3_	0.69, s	13.5	0.74, s	13.1
COCH_3_			2.04, s	21.1
COCH_3_				172.8

*^a^*^1^H NMR recorded at 400 MHz, ^13^C NMR recorded at 100 MHz.

## Supplementary Materials

The following are available online at https://www.mdpi.com/1660-3397/18/3/164/s1, Figures S1–S37: ^1^H, ^13^C NMR, HSQC, ^1^H-^1^H COSY, HMBC, HRESIMS spectra of the new compounds **1**, **9**, and **10**, ^1^H and ^13^C NMR of known compounds **2**−**8**, and **11****,** and details for ECD calculations.
