# Supplementary material for: Terpenoids from the Deep-Sea-Derived Fungus Penicillium thomii YPGA3 and Their Bioactivities
Source: Mar Drugs. 2020 Mar 16;18(3):164. doi: 10.3390/md18030164 (PMC7143578; doi:10.3390/md18030164)
Supplement: Supplementary file 1 [file marinedrugs-18-00164-s001.pdf]

Terpenoids from the Deep-Sea-Derived Fungus *Penicillium thomii* YPGA3 and Their Bioactivities

Zhongbin Cheng [1,4,*], Wan Liu [1], Runzhu Fan [3], Shouye Han [1], Yuanli Li [1], Xiaoyun Cui [1], Jia Zhang [1], Yinan Wu [1], Xin Lv [1], Yun Zhang [1], Zhuhua Luo [2], Siti Aisyah Alias [5], Wei Xu [2,*] and Qin Li [1,4,*]

[1] School of Pharmacy, Henan University, Kaifeng 475004, China; 18737806806@163.com (W.L.); hanshouye123@163.com (S.H.); lyl3287439993@163.com (Y.L.); cxy521bjt@163.com (X.C.); z919395@126.com (J.Z.); w1287457895@163.com (Y.W.); 15225826138@163.com (X.L); zy190831@163.com (Y.Z.)
[2] Key Laboratory of Marine Biogenetic Resources, Third Institute of Oceanography, Ministry of Natural Resources, Xiamen 361005, China; luozhuhua@tio.org.cn (Z.L.)
[3] School of Pharmaceutical Sciences, Sun Yat-sen University, Guangzhou 510006, China, fanrzh@mail2.sysu.edu.cn (R.F.)
[4] Eucommia Ulmoides Cultivation and Utilization of Henan Engineering Laboratory, Kaifeng 475004, China
[5] Institute of Ocean and Earth Science (IOES), C308, Institute of Postgraduate Studies Building, University of Malaya, 50603 Kuala Lumpur, Malaysia; siti.alias@gmail.con (A.S.A.)
* Correspondence: czb360@126.com (Z.C.); xuwei@tio.org.cn (W.X.); liqin6006@163.com (Q.L.); Tel.: +86-371-2388-3849 (Q.L.)

Table of Contents		Page
**Figure S1**	^{1}H NMR Spectrum of **1** in Methanol-*d*4 (400 MHz).	1
**Figure S2**	^{13}C NMR Spectrum of **1** in Methanol-*d*4 (100 MHz)	1
**Figure S3**	HSQC Spectrum of **1** in Methanol-*d*4	2
**Figure S4**	^{1}H-^{1}H COSY Spectrum of **1** in Methanol-*d*4	2
**Figure S5**	HMBC Spectrum of **1** in Methanol-*d*4	3
**Figure S6**	NOESY Spectrum of **1** in Methanol-*d*4	3
**Figure S7**	^{1}H NMR Spectrum of **9** in Methanol-*d*4 (400 MHz)	4
**Figure S8**	^{13}C NMR Spectrum of **9** in Methanol-*d*4 (100 MHz)	4
**Figure S9**	HSQC Spectrum of **9** in Methanol-*d*4	5
**Figure S10**	^{1}H-^{1}H COSY Spectrum of **9** in Methanol-*d*4	5
**Figure S11**	HMBC Spectrum of **9** in Methanol-*d*4	6
**Figure S12**	NOESY Spectrum of **9** in Methanol-*d*4	6
**Figure S13**	^{1}H NMR Spectrum of **10** in Methanol-*d*4 (400 MHz)	7
**Figure S14**	^{13}C NMR Spectrum of **10** in Methanol-*d*4 (100 MHz)	7
**Figure S15**	HSQC Spectrum of **10** in Methanol-*d*4	8
**Figure S16**	^{1}H-^{1}H COSY Spectrum of **10** in Methanol-*d*4	8

**Figure S17**	HMBC Spectrum of **10** in Methanol-d_4	9
**Figure S18**	NOESY Spectrum of **10** in Methanol-d_4	9
**Figure S19**	^{1}H NMR Spectrum of **2** in Methanol-d_4	10
**Figure S20**	^{13}C NMR Spectrum of **2** in Methanol-d_4	10
**Figure S21**	^{1}H NMR Spectrum of **3** in Methanol-d_4	11
**Figure S22**	^{13}C NMR Spectrum of **3** in Methanol-d_4	11
**Figure S23**	^{1}H NMR Spectrum of **4** in Methanol-d_4	12
**Figure S24**	^{13}C NMR Spectrum of **4** in Methanol-d_4	12
**Figure S25**	^{1}H NMR Spectrum of **5** in Methanol-d_4	13
**Figure S26**	^{13}C NMR Spectrum of **5** in Methanol-d_4	13
**Figure S27**	^{1}H NMR Spectrum of **6** in Methanol-d_4	14
**Figure S28**	^{13}C NMR Spectrum of **6** in Methanol-d_4	14
**Figure S29**	^{1}H NMR Spectrum of **7** in Methanol-d_4	15
**Figure S30**	^{13}C NMR Spectrum of **7** in Methanol-d_4	15
**Figure S31**	^{1}H NMR Spectrum of **8** in Methanol-d_4	16
**Figure S32**	^{1}H NMR Spectrum of **11** in Methanol-d_4	16
**Figure S33**	^{13}C NMR Spectrum of **11** in Methanol-d_4	17
**Figure S34**	HRESIMS spectrum of **1**	17
**Figure S35**	HRESIMS spectrum of **9**	18
**Figure S36**	HRESIMS spectrum of **10**	18
**S37**	Details for ECD calculations of **1** and **9**	19
**Table S1**	Inhibitions (%) on NO production and cytotoxicity (%) toward RAW 264.7, MCF-7, and MDA-MB-468 cells (cell viability) of compounds **1**−**11**.	23

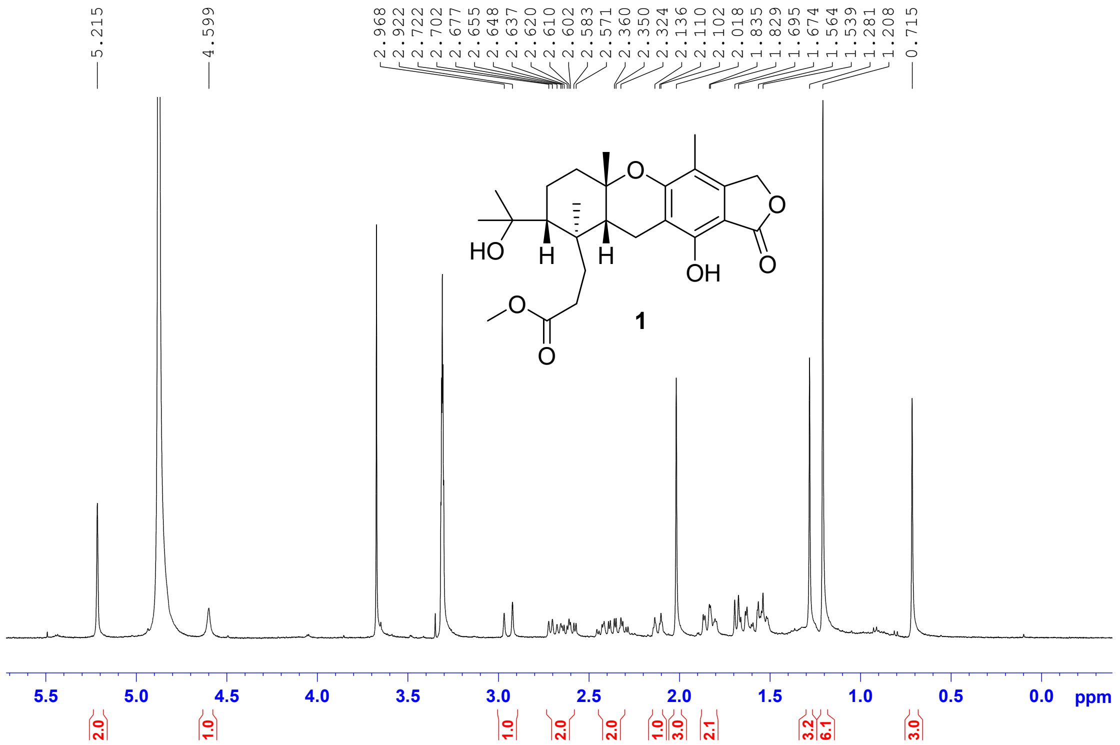

Figure S1 ^{1}H NMR Spectrum of **1** in Methanol-d_4 (400 MHz)

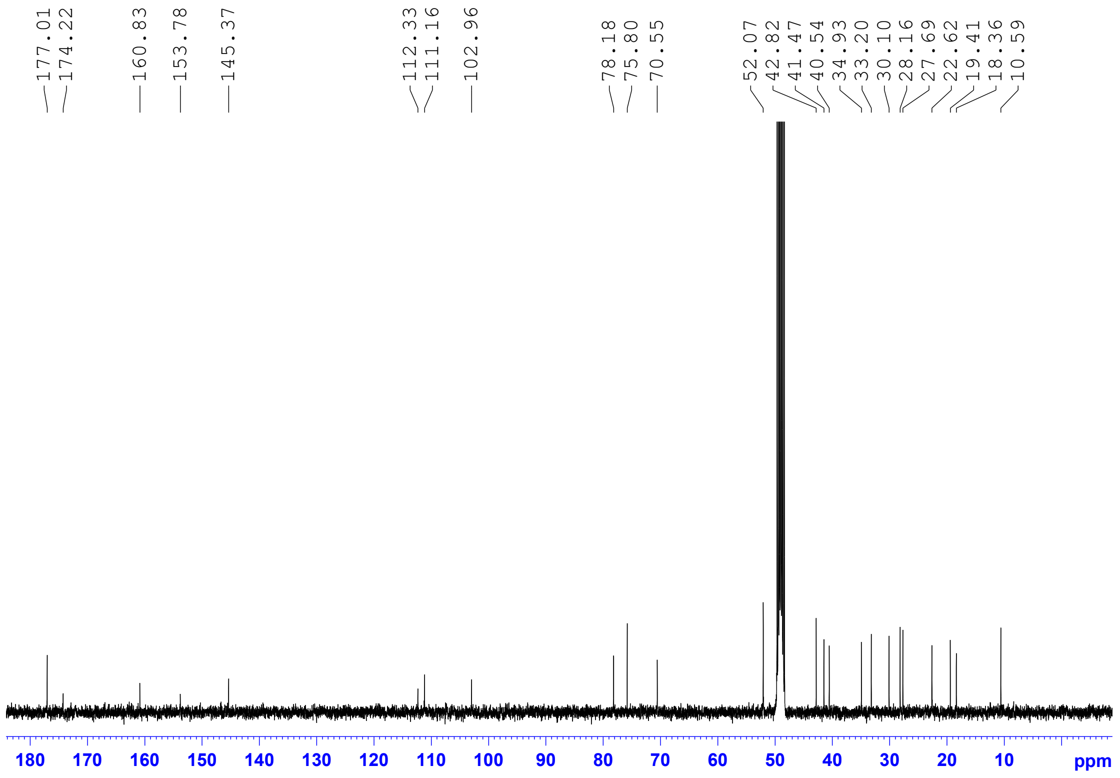

Figure S2 ^{13}C NMR Spectrum of **1** in Methanol-d_4 (100 MHz)

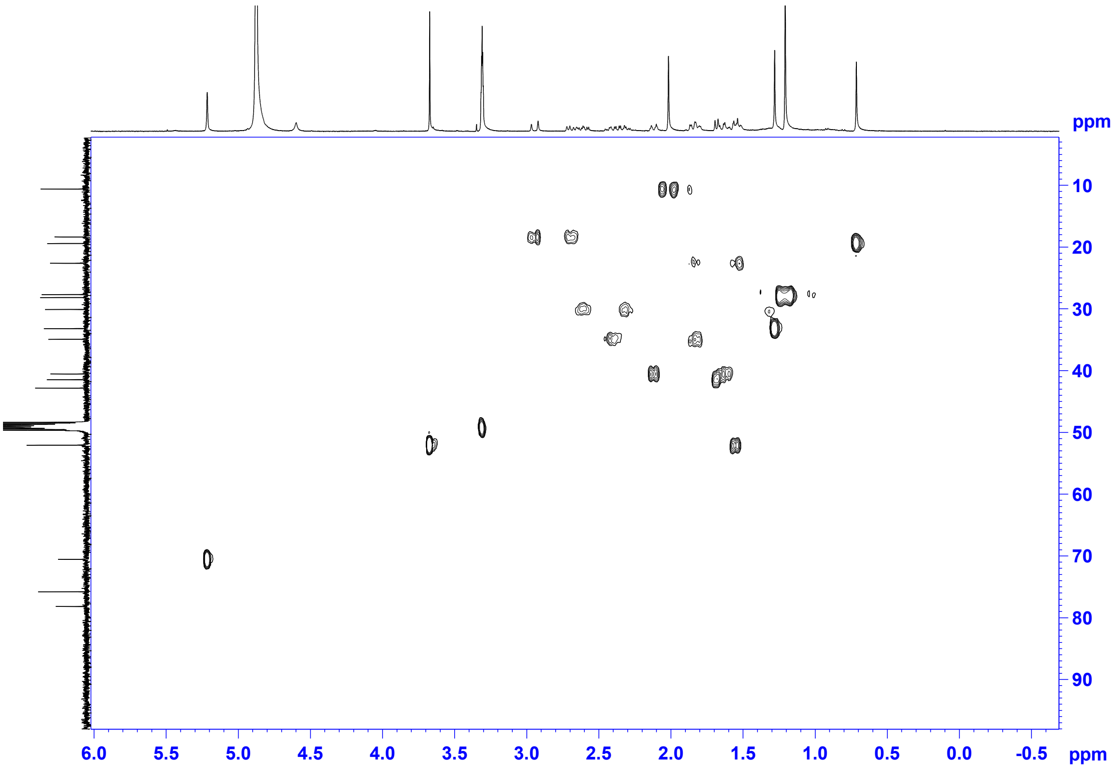

Figure S3 HSQC Spectrum of **1** in Methanol-d_4

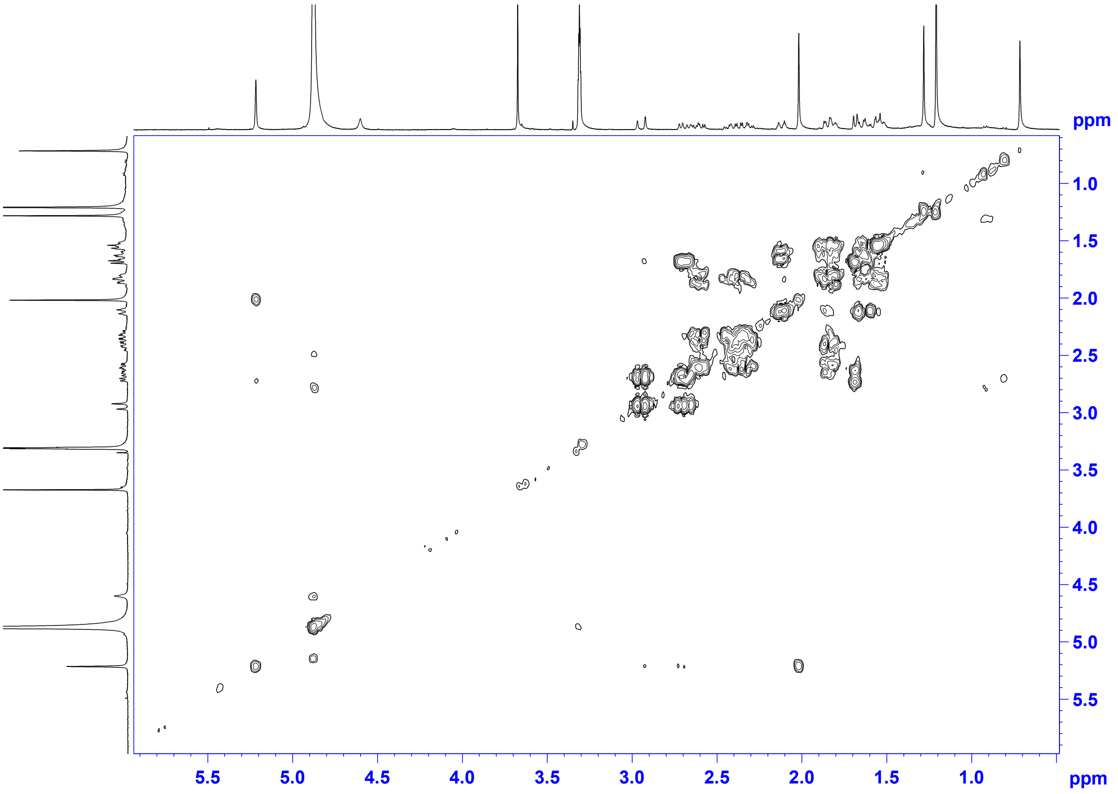

Figure S4 ^{1}H-^{1}H COSY Spectrum of **1** in Methanol-d_4

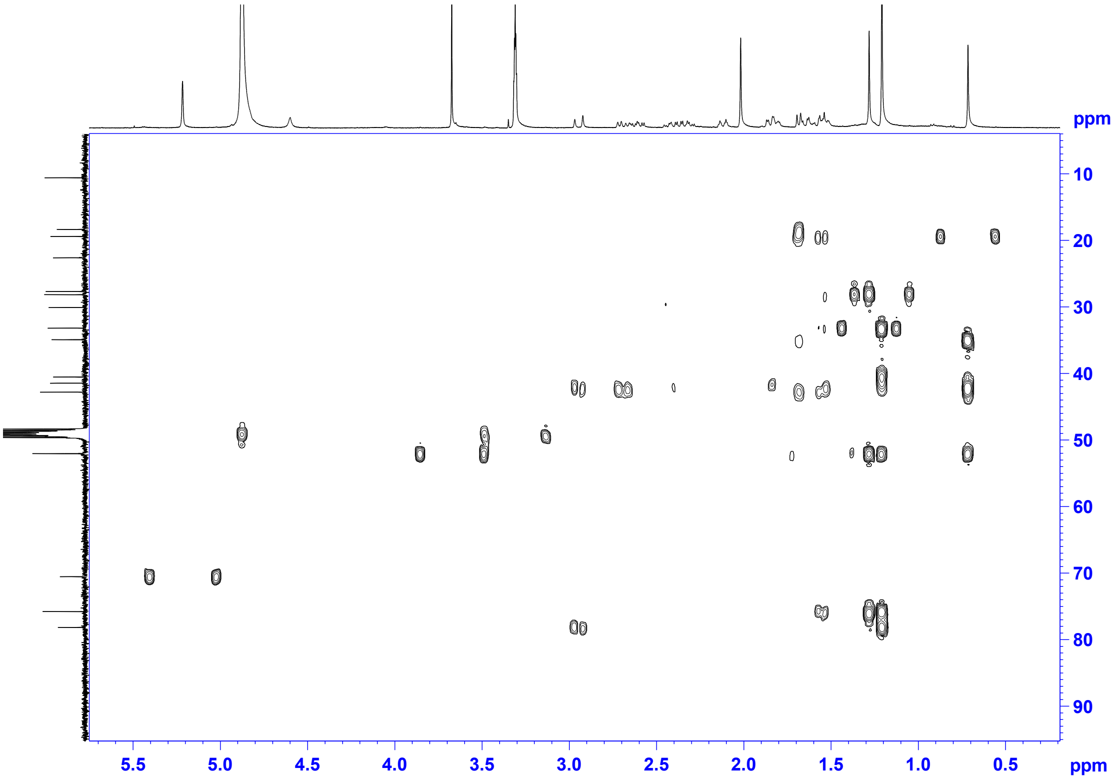

Figure S5 HMBC Spectrum of **1** in Methanol-d_4

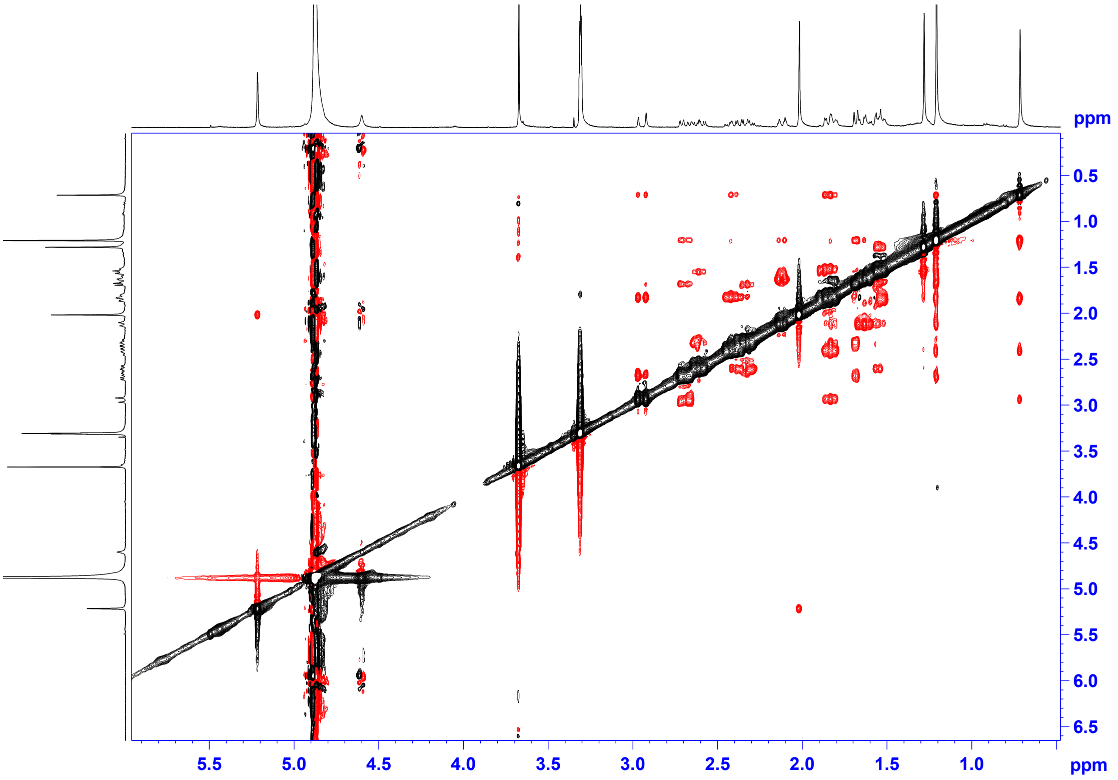

Figure S6 NOESY Spectrum of **1** in Methanol-d_4

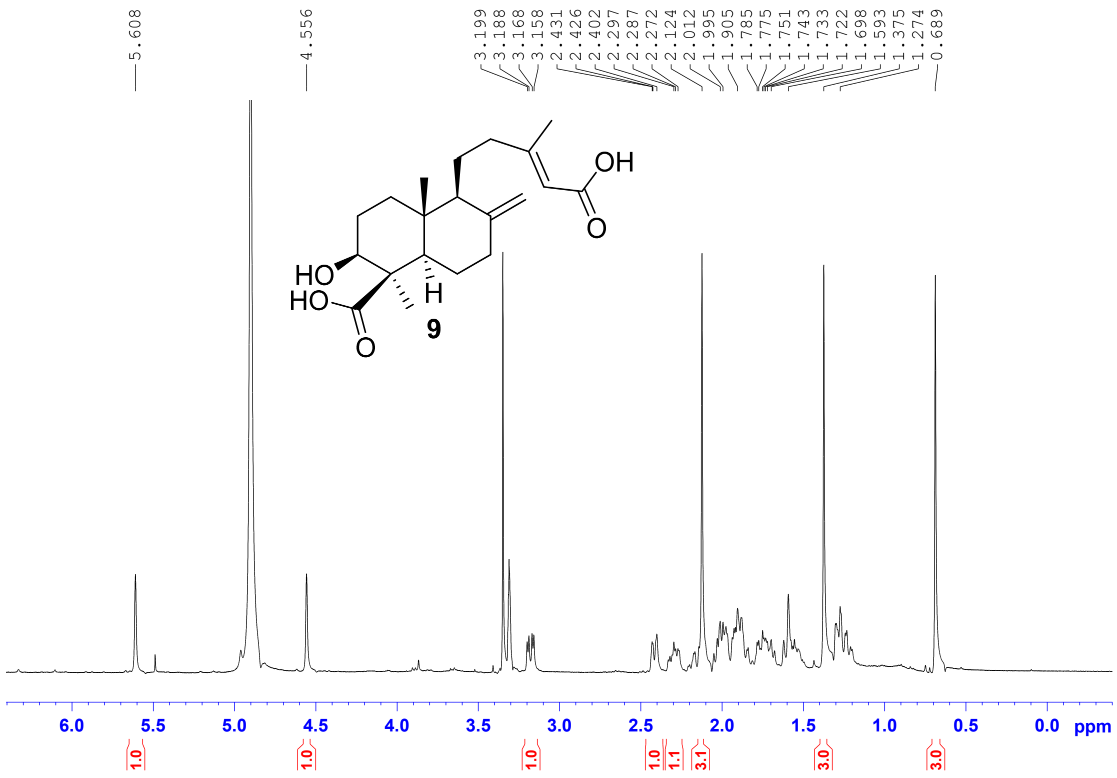

Figure S7 ^{1}H NMR Spectrum of **9** in Methanol-*d*4 (400 MHz)

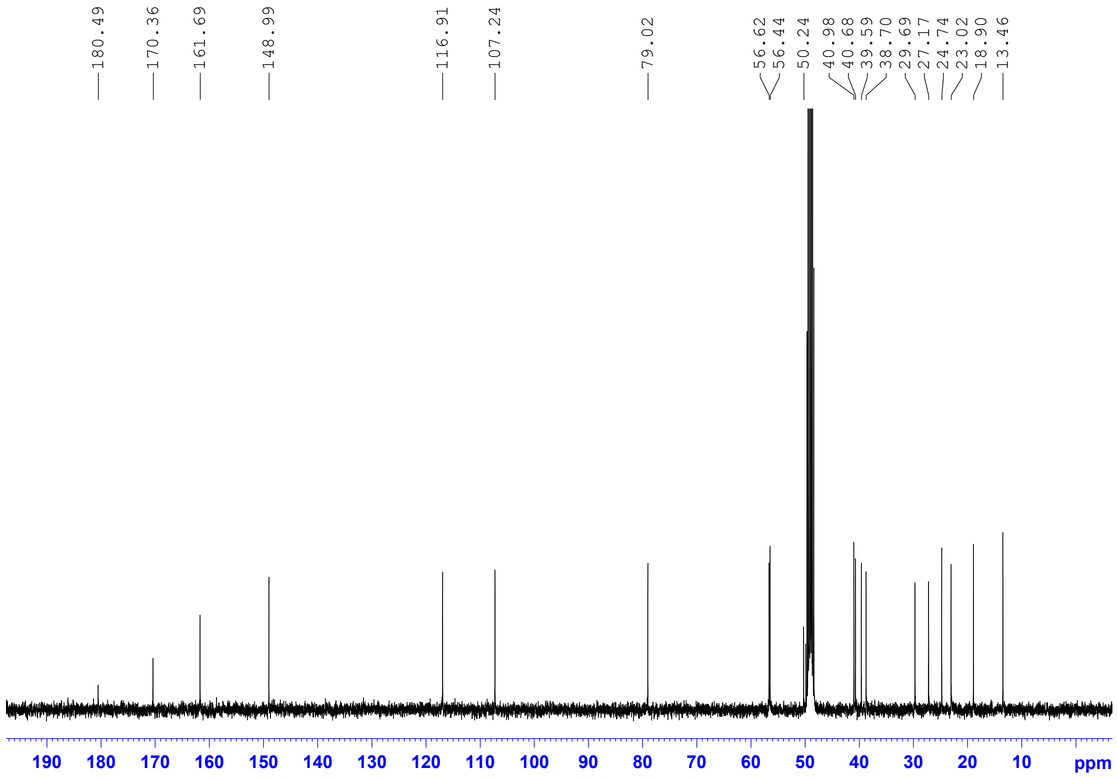

Figure S8 ^{13}C NMR Spectrum of **9** in Methanol-*d*4 (100 MHz)

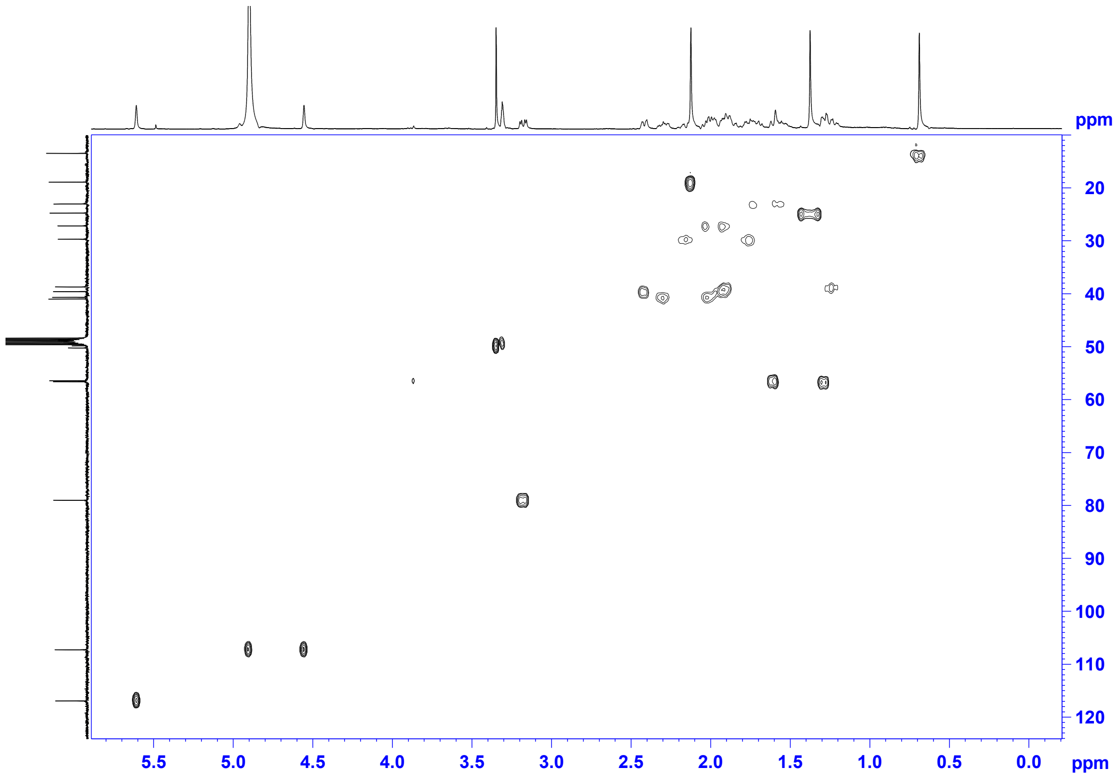

Figure S9 HSQC Spectrum of **9** in Methanol-d_4

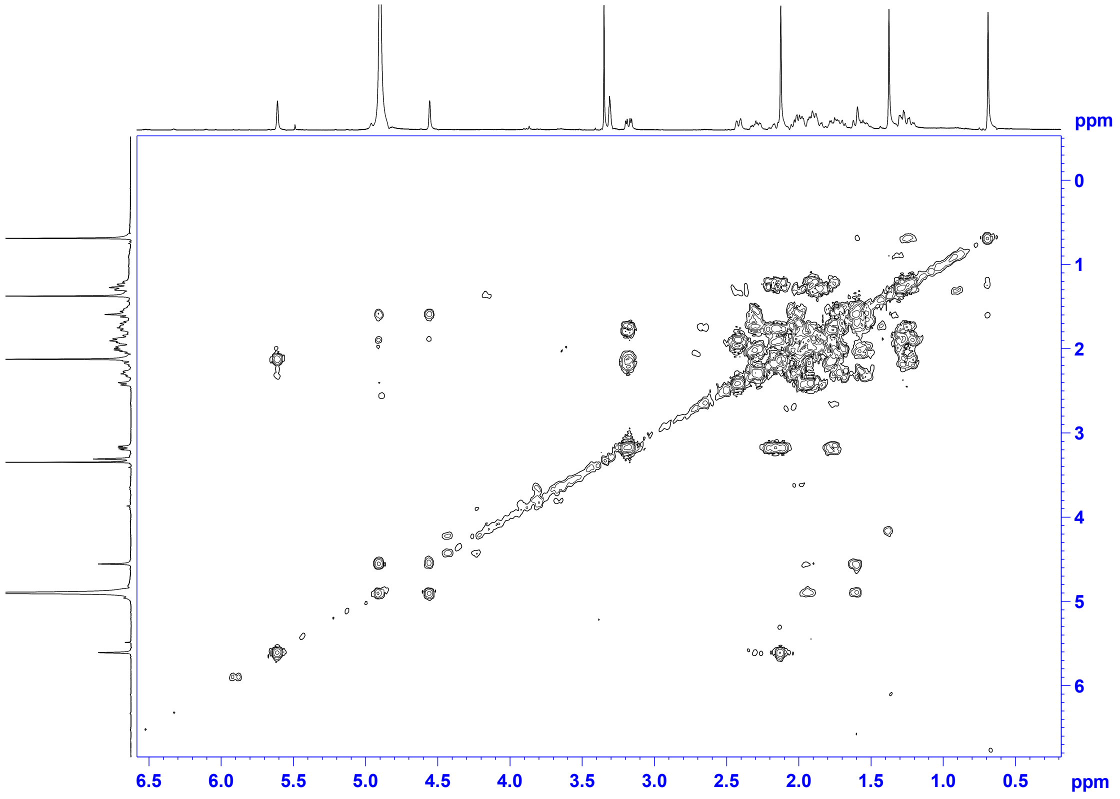

Figure S10 ^{1}H-^{1}H COSY Spectrum of **9** in Methanol-d_4

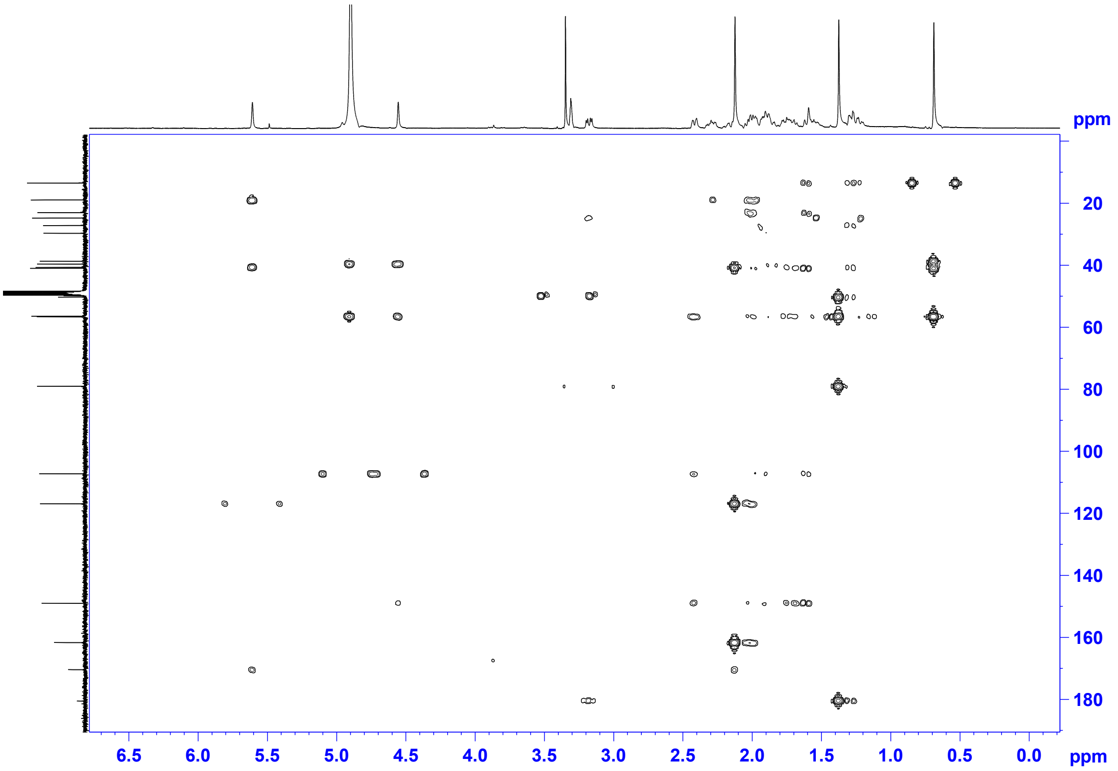

Figure S11 HMBC Spectrum of **9** in Methanol-*d*4

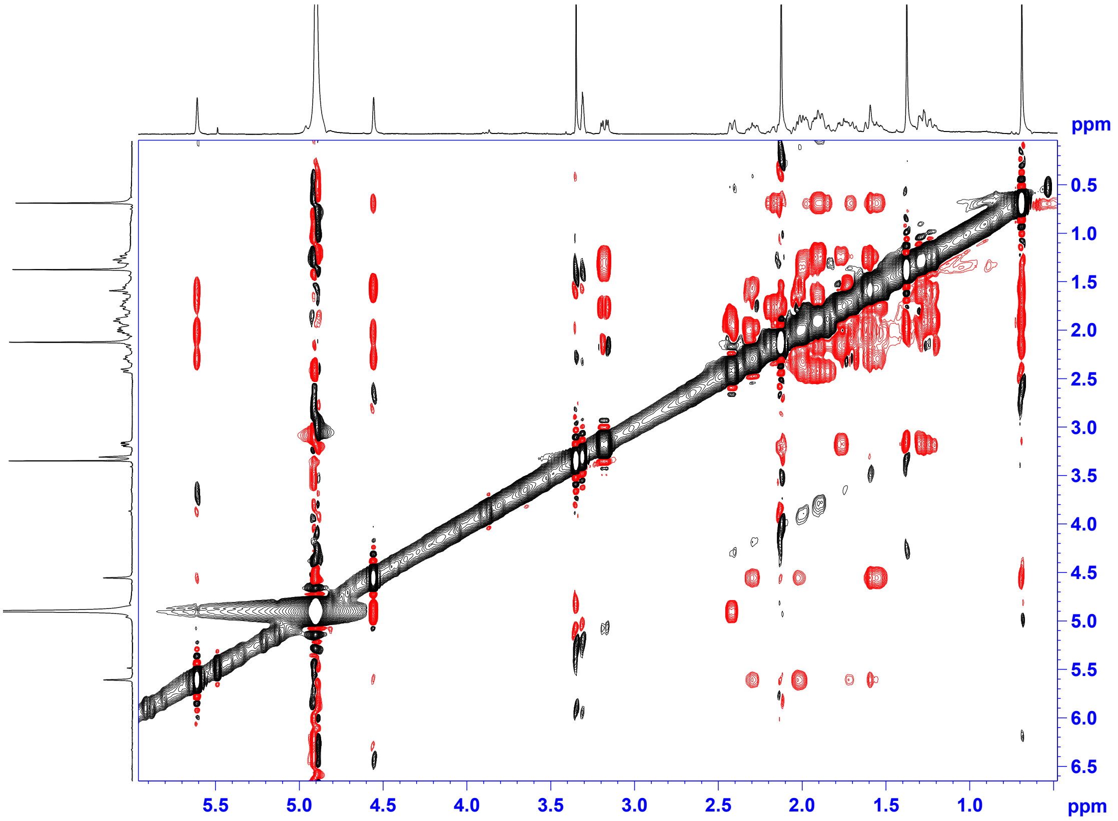

Figure S12 NOESY Spectrum of **9** in Methanol-*d*4

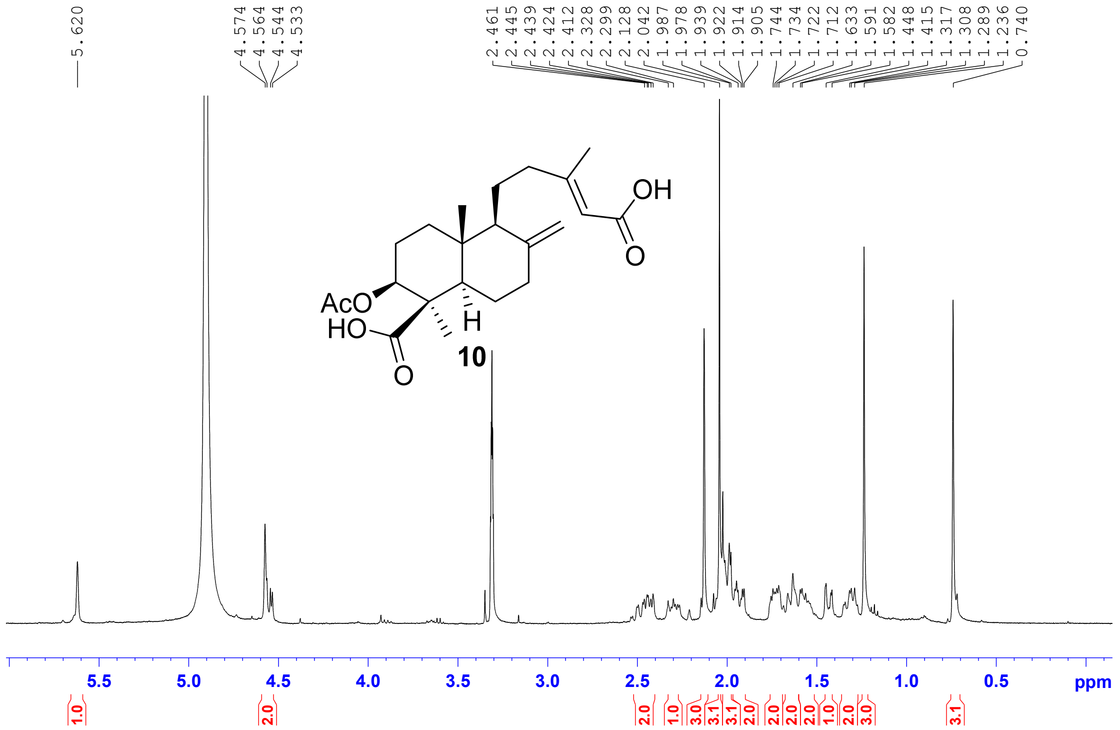

Figure S13 ^{1}H NMR Spectrum of **10** in Methanol-*d*4 (400 MHz).

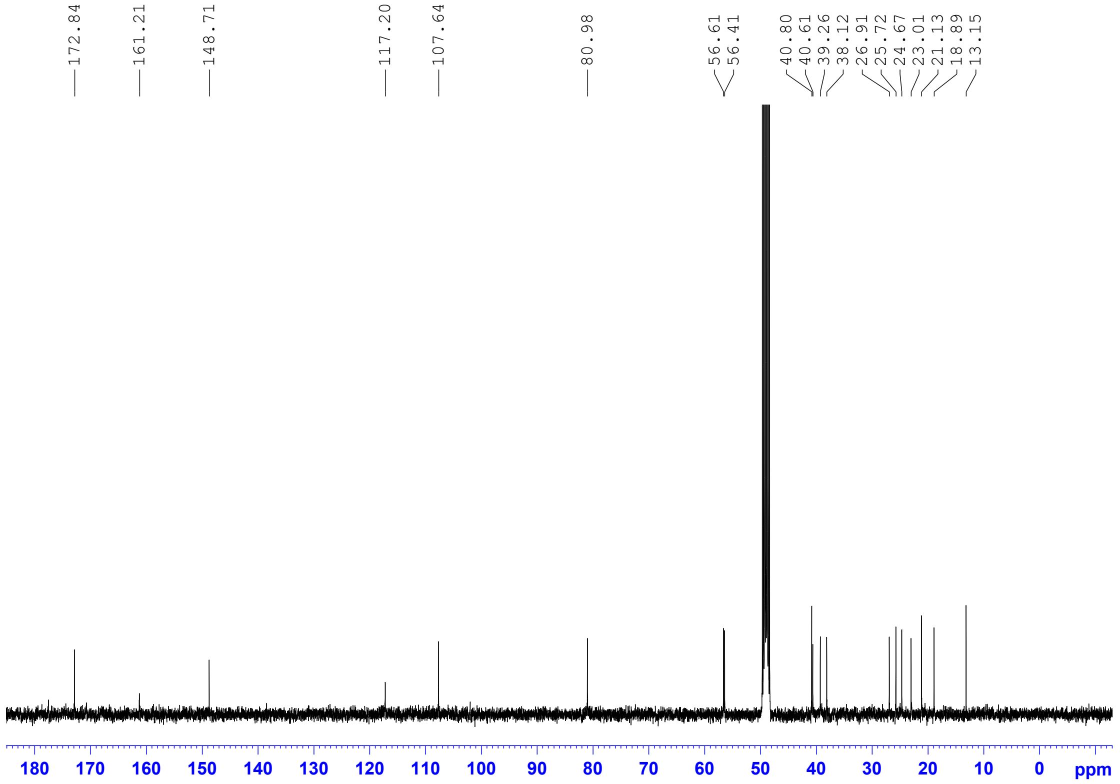

Figure S14 ^{13}C NMR Spectrum of **10** in Methanol-*d*4 (100 MHz)

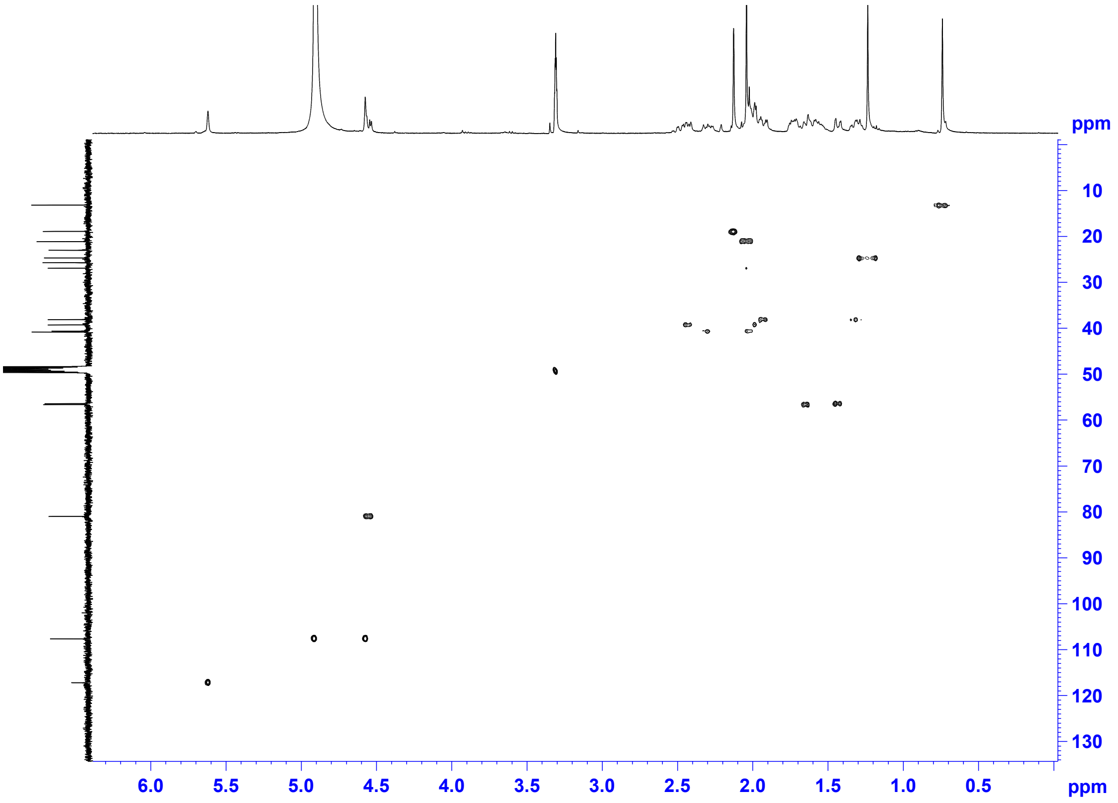

Figure S15 HSQC Spectrum of **10** in Methanol-*d*4

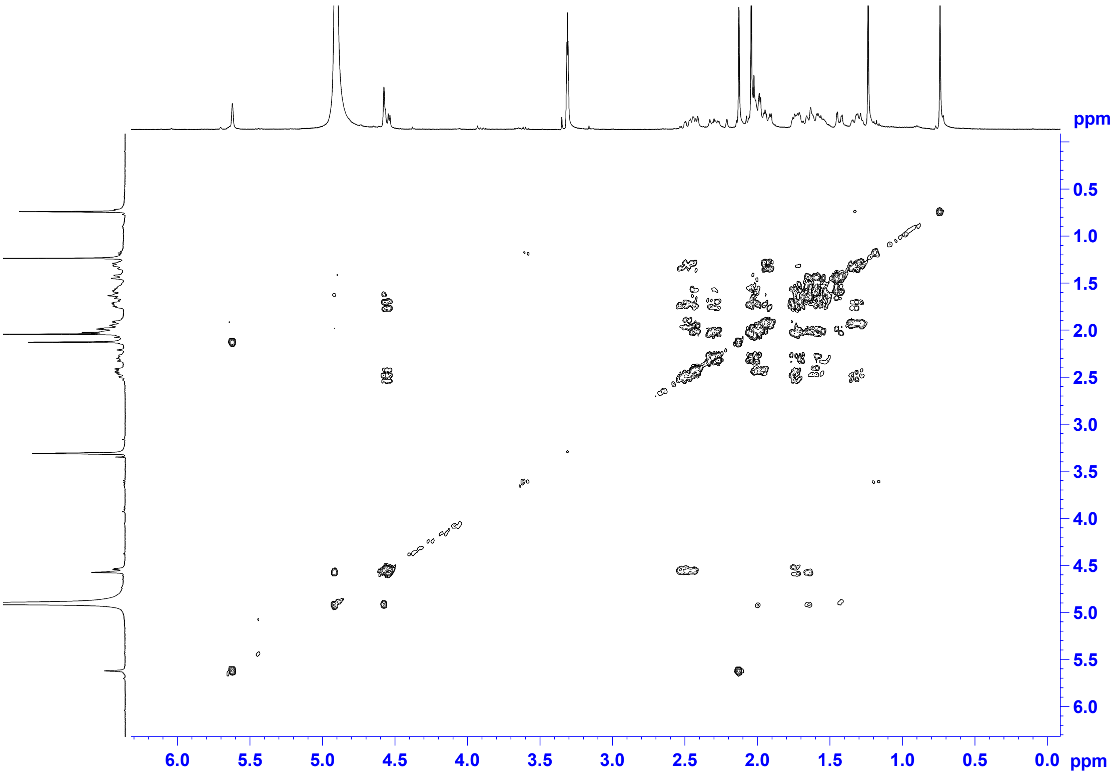

Figure S16 ^{1}H-^{1}H COSY Spectrum of **10** in Methanol-*d*4

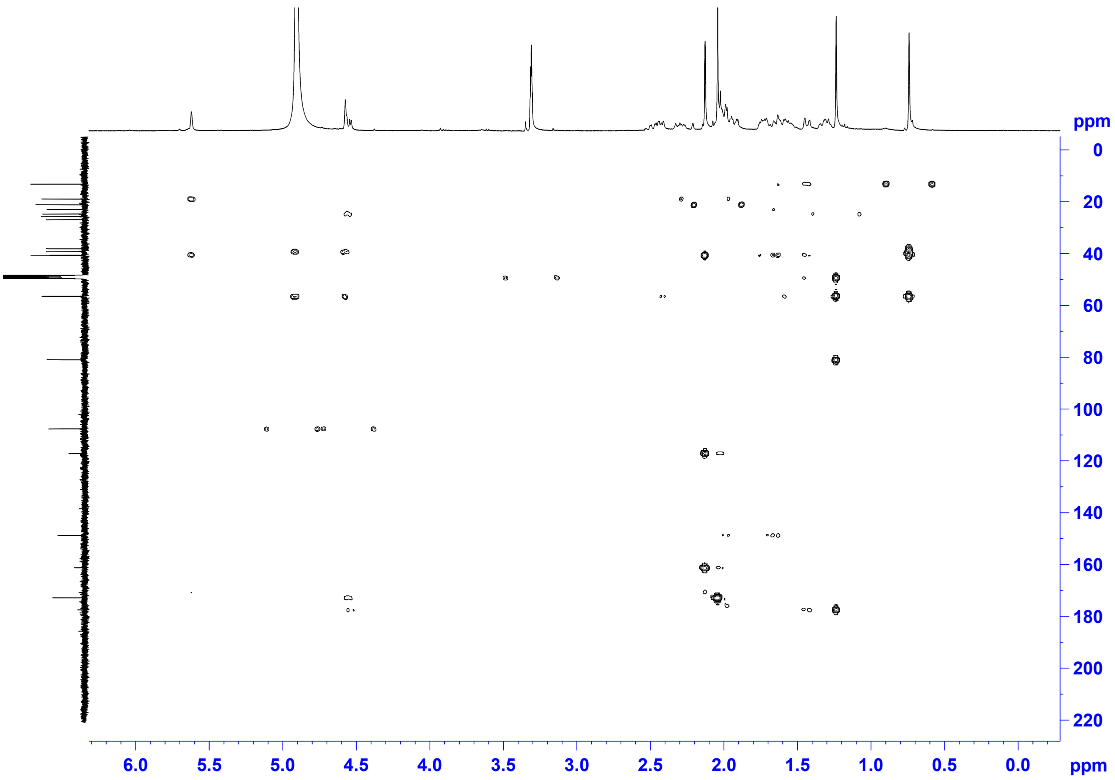

Figure S17 HMBC Spectrum of **10** in Methanol-d_4

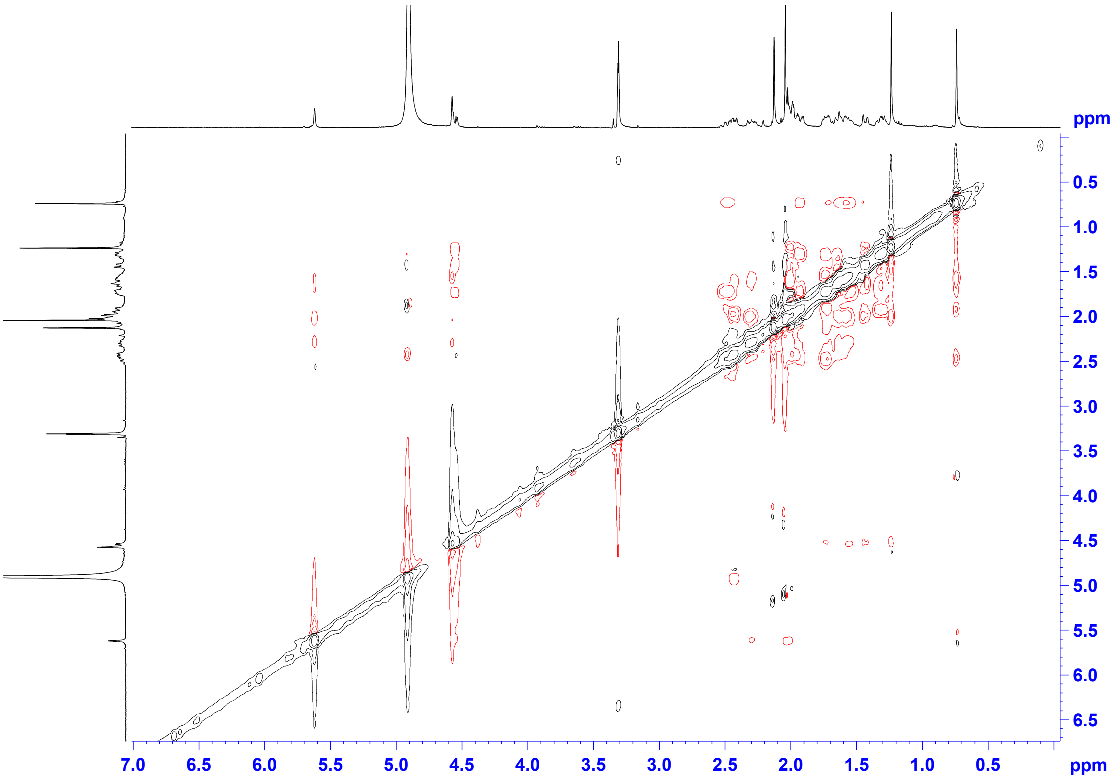

Figure S18 NOESY Spectrum of **10** in Methanol-d_4

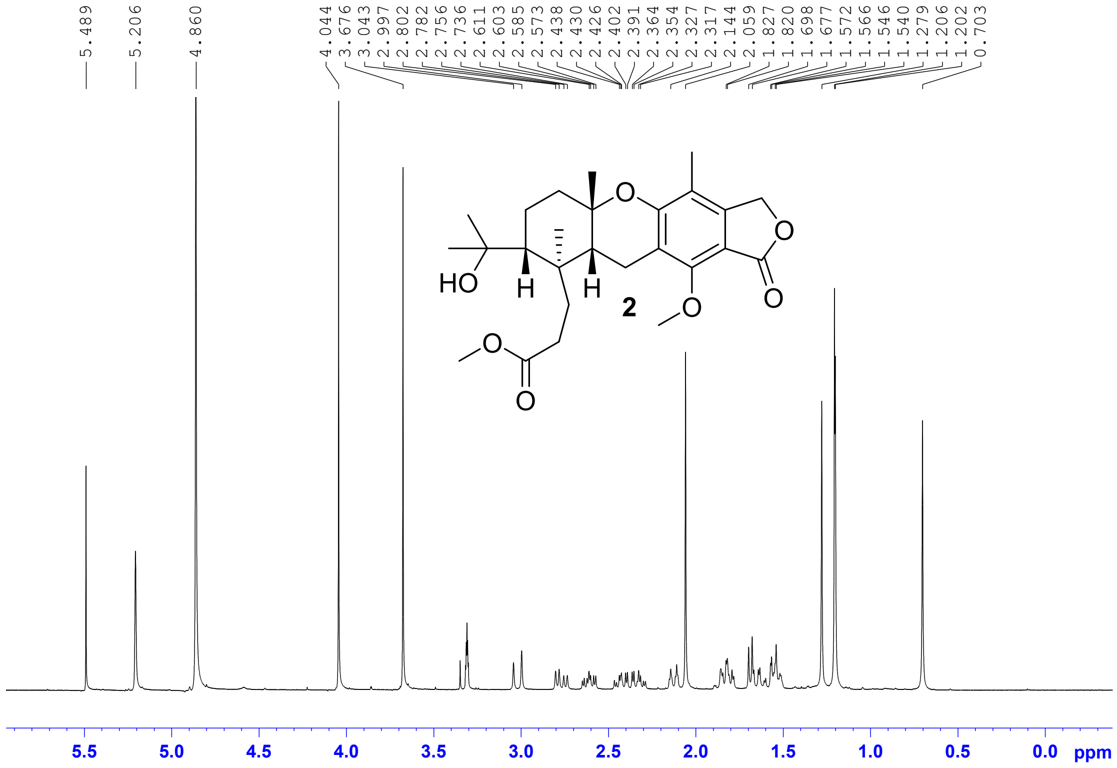

Figure S19 ^{1}H NMR Spectrum of **2** in Methanol-*d*4

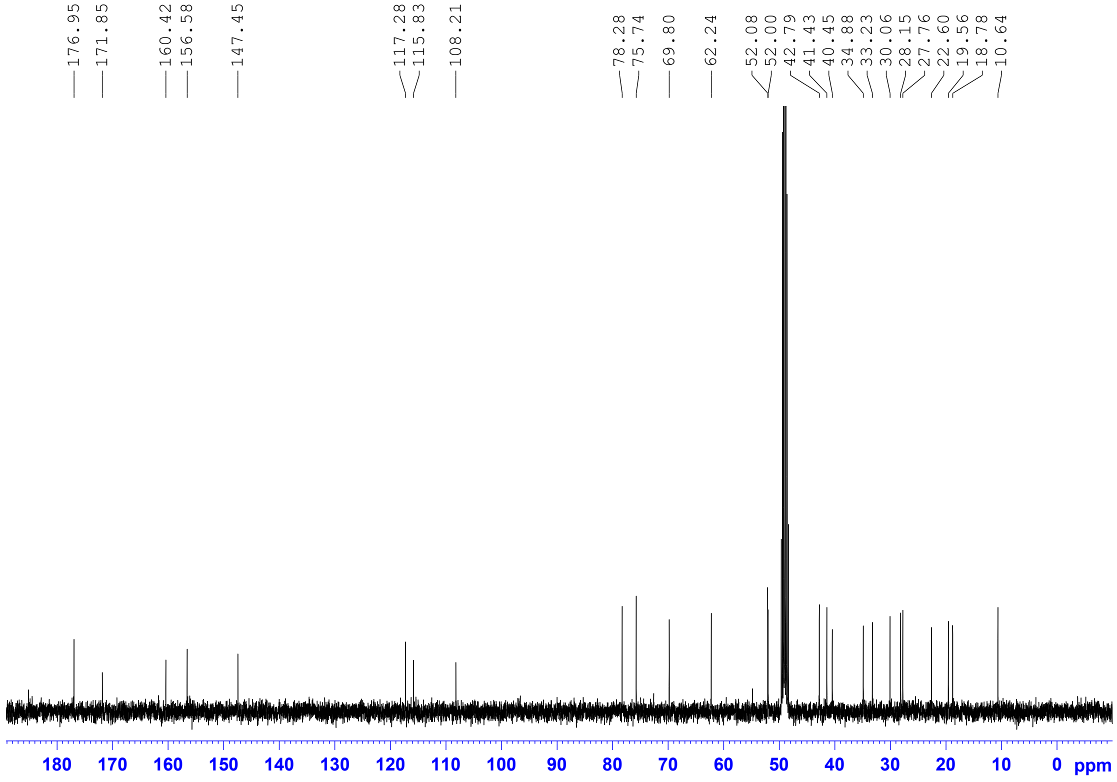

Figure S20 ^{13}C NMR Spectrum of **2** in Methanol-*d*4

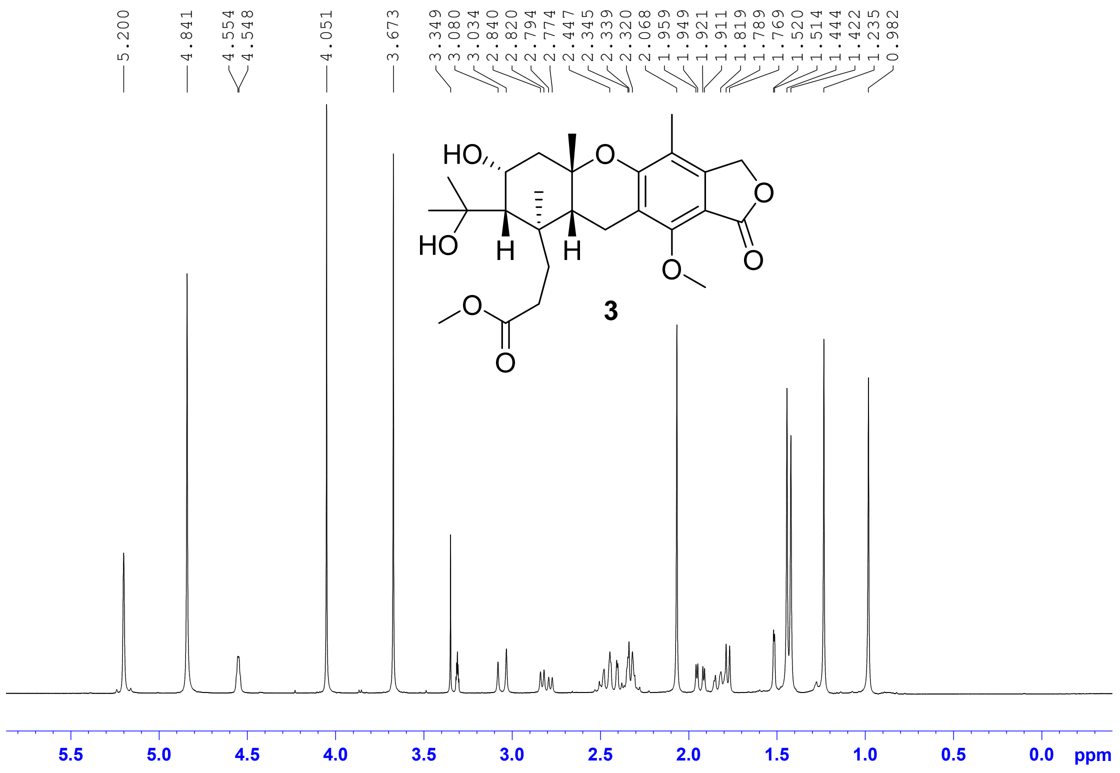

Figure S21 ^{1}H NMR Spectrum of **3** in Methanol-d_4

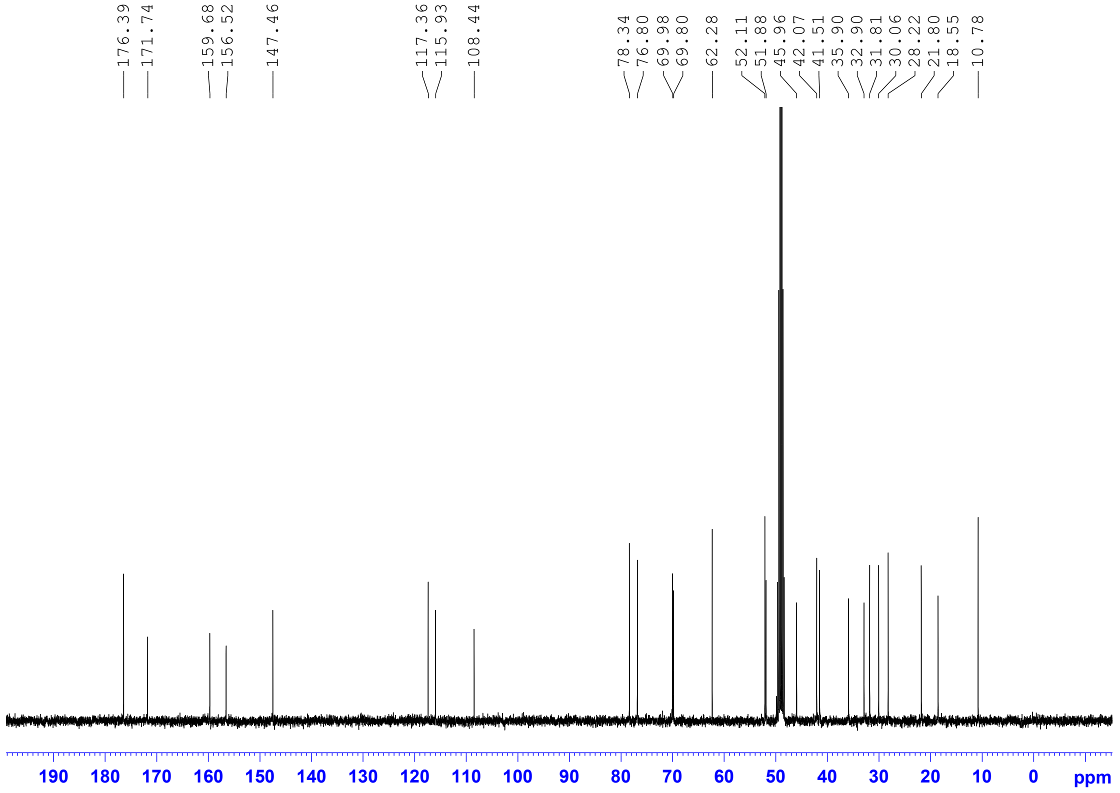

Figure S22 ^{13}C NMR Spectrum of **3** in Methanol-d_4

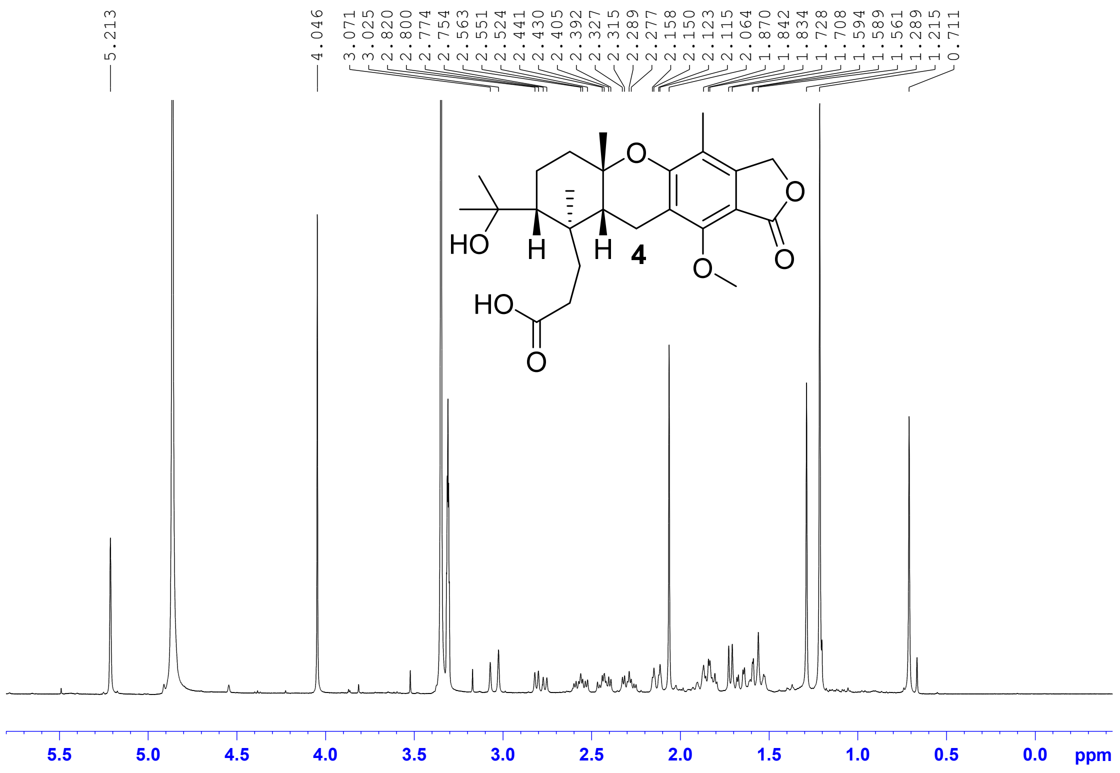

Figure S23 ^{1}H NMR Spectrum of **4** in Methanol-d_4

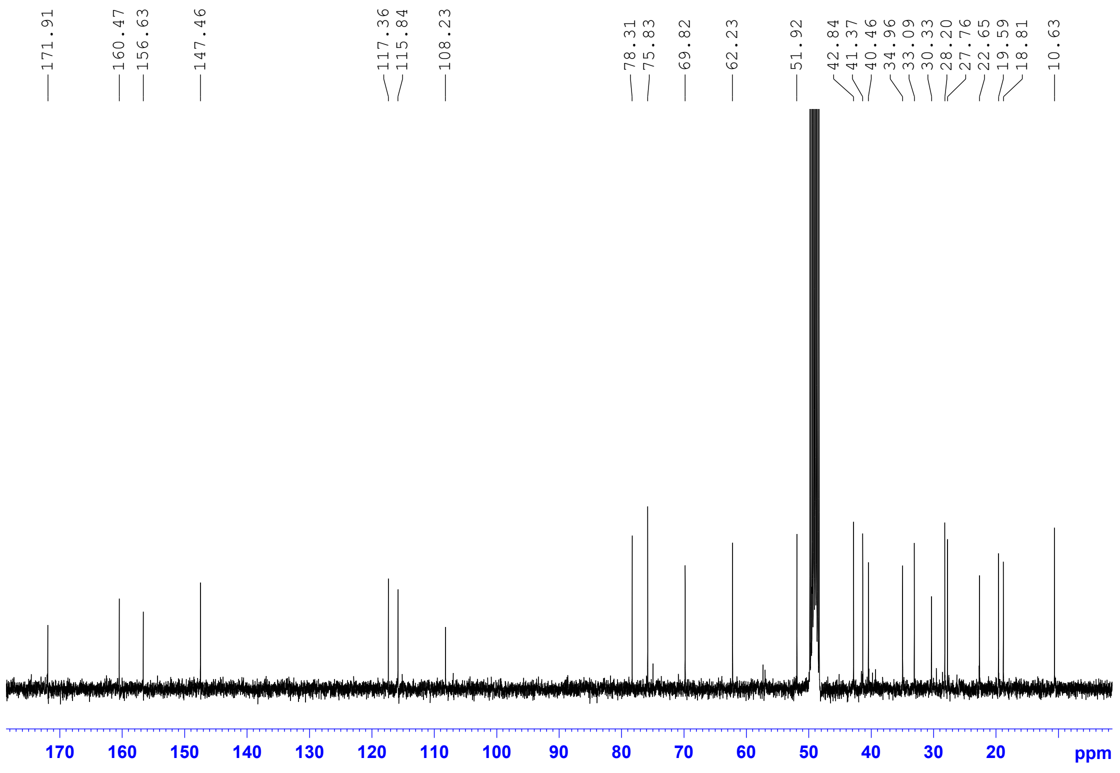

Figure S24 ^{13}C NMR Spectrum of **4** in Methanol-d_4

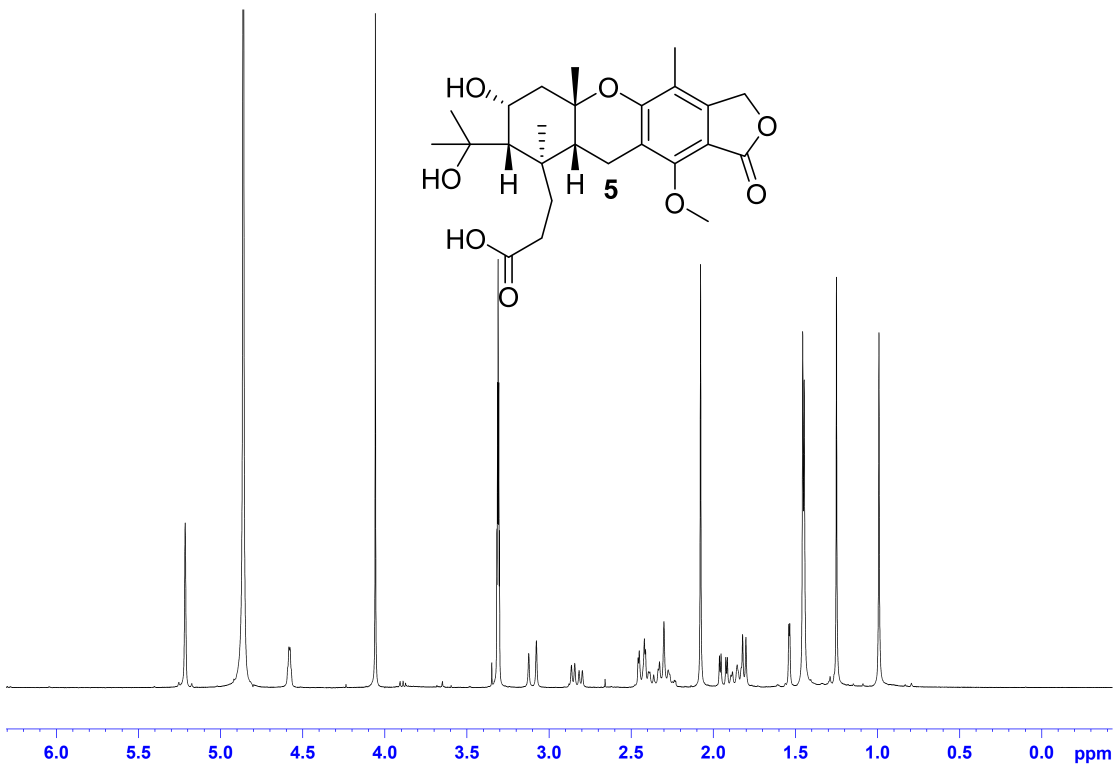

Figure S25 ^{1}H NMR Spectrum of **5** in Methanol-*d*4

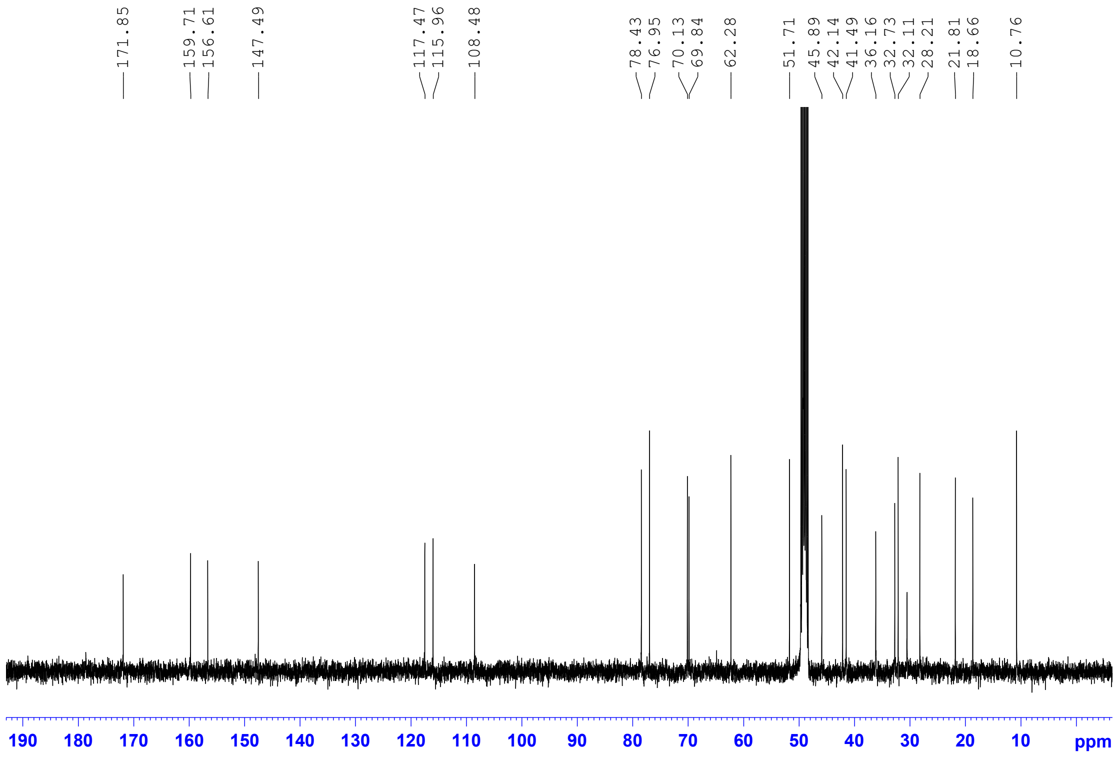

Figure S26 ^{13}C NMR Spectrum of **5** in Methanol-*d*4

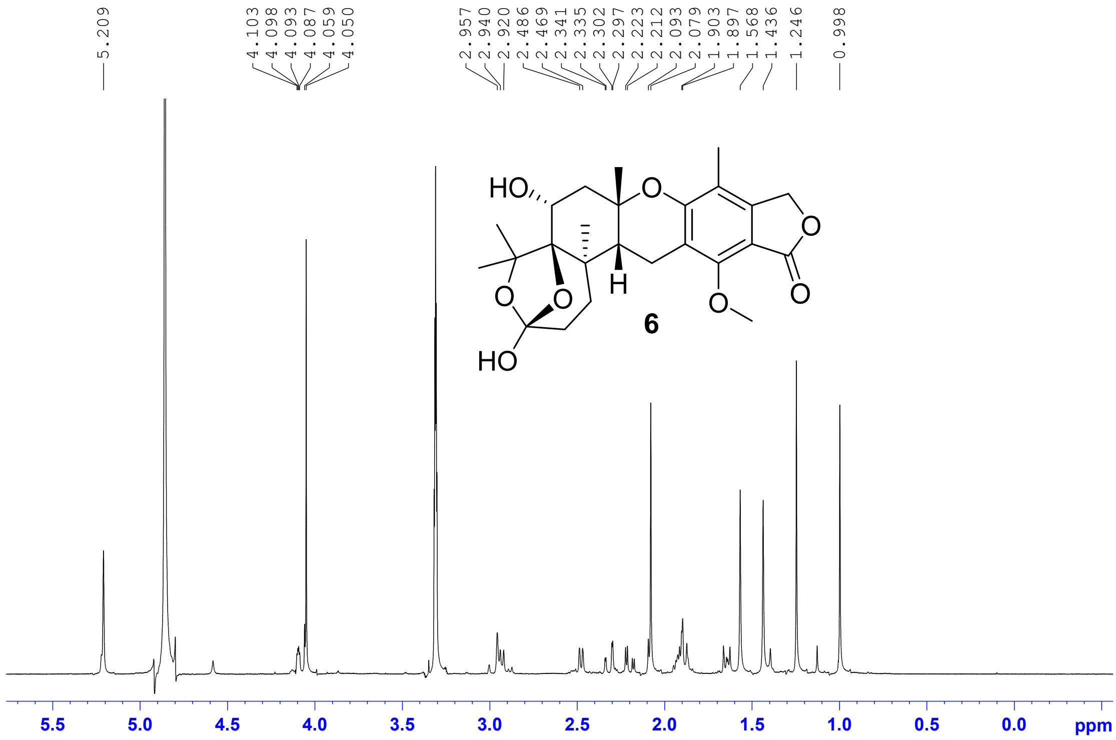

Figure S27 ^{1}H NMR Spectrum of **6** in Methanol-*d*4

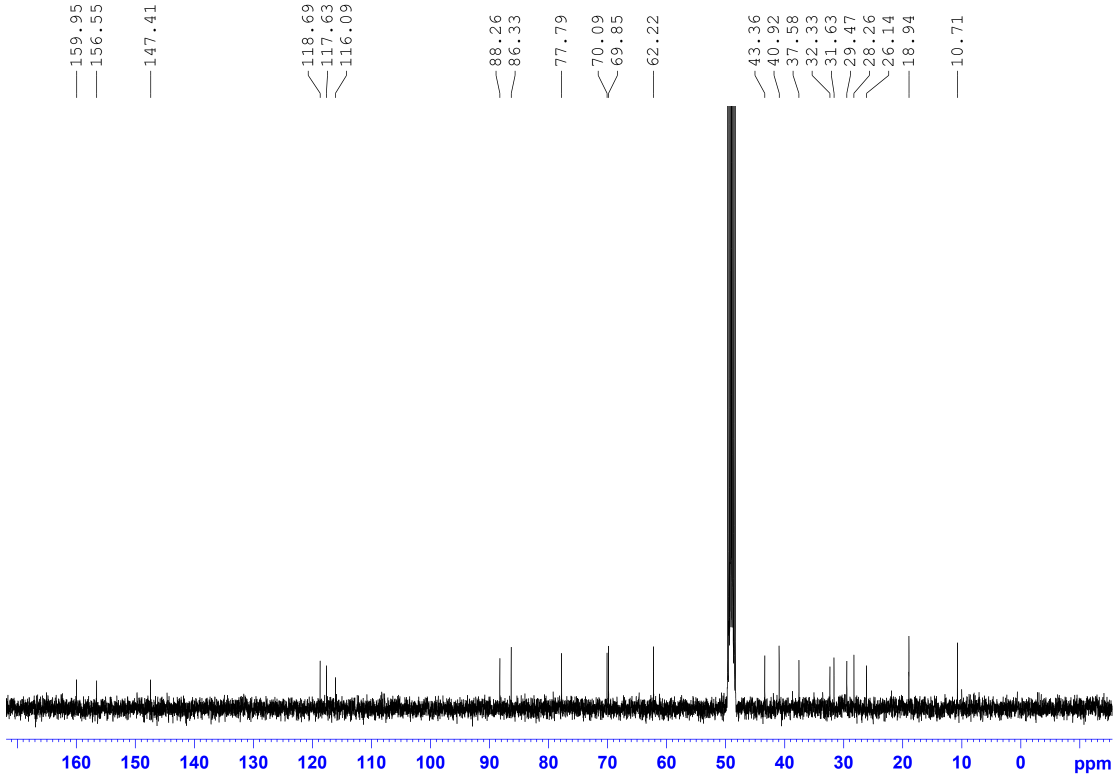

Figure S28 ^{13}C NMR Spectrum of **6** in Methanol-*d*4

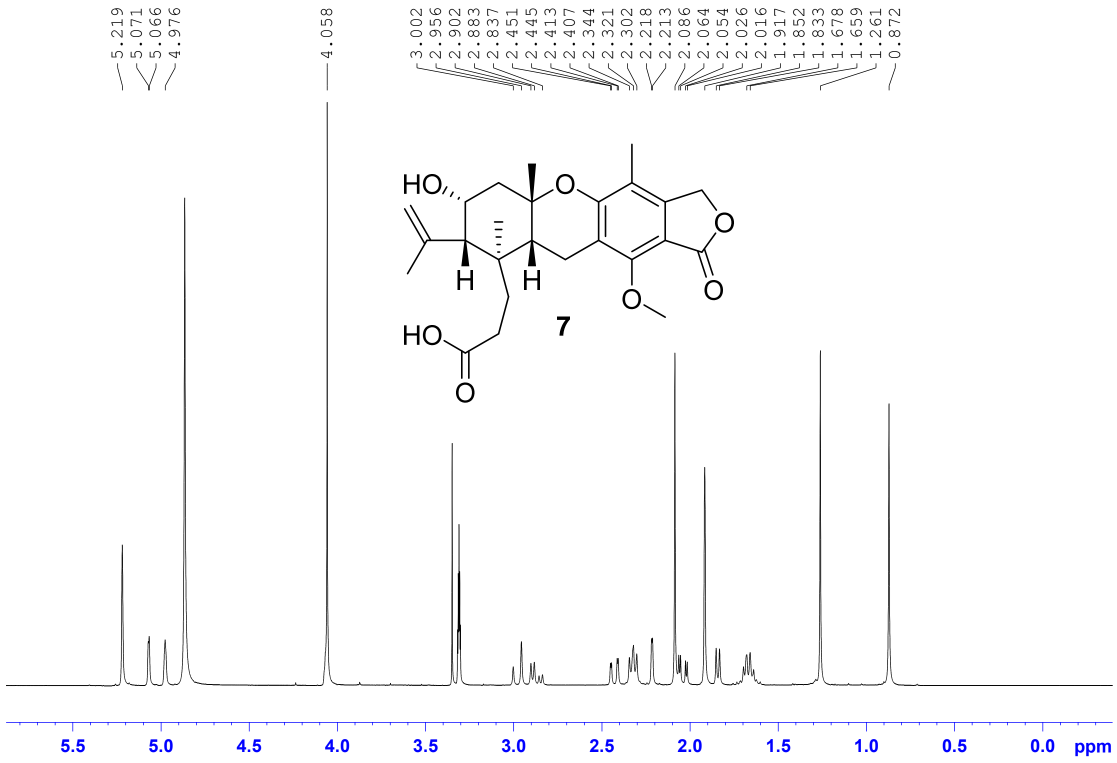

Figure S29 ^{1}H NMR Spectrum of **7** in Methanol-d_4

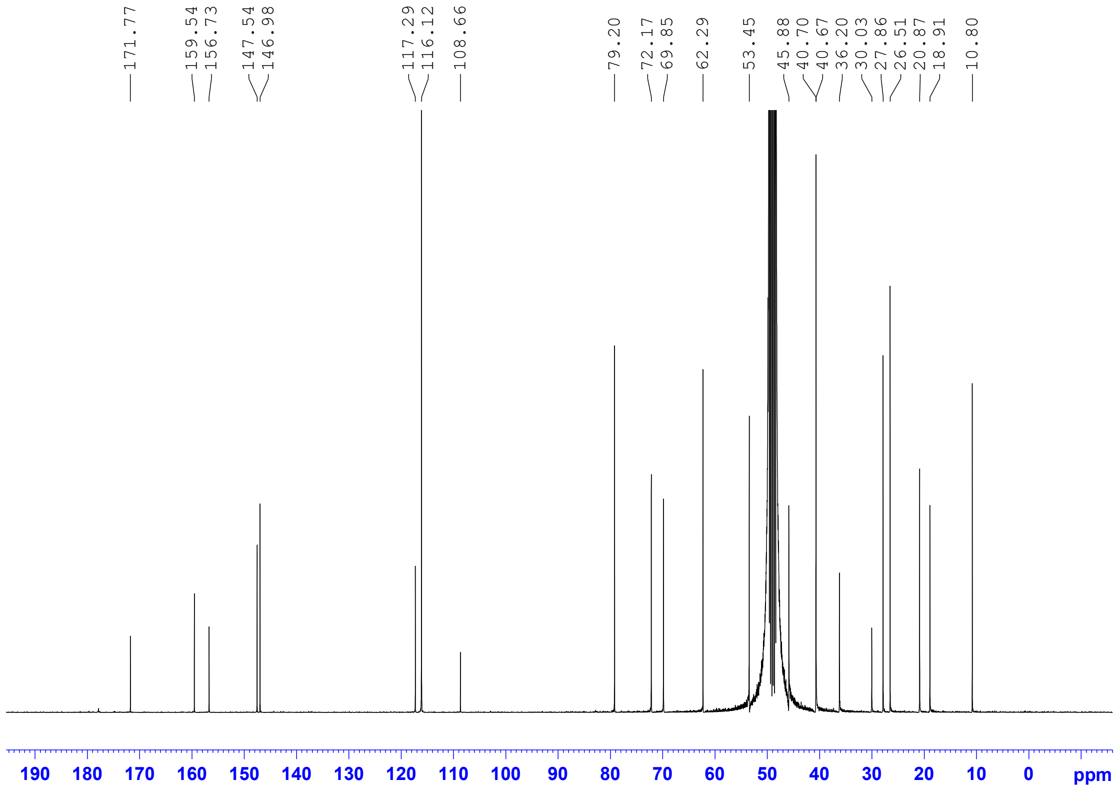

Figure S30 ^{13}C NMR Spectrum of **7** in Methanol-d_4

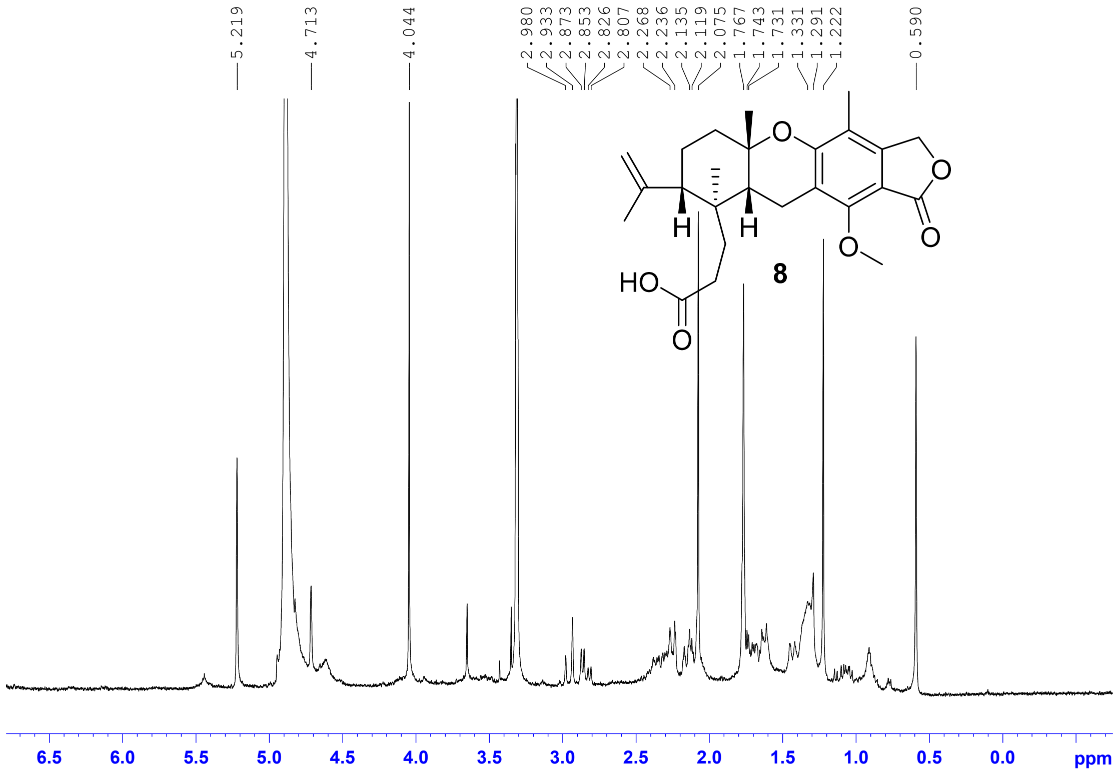

Figure S31 ^{1}H NMR Spectrum of **8** in Methanol-d_4

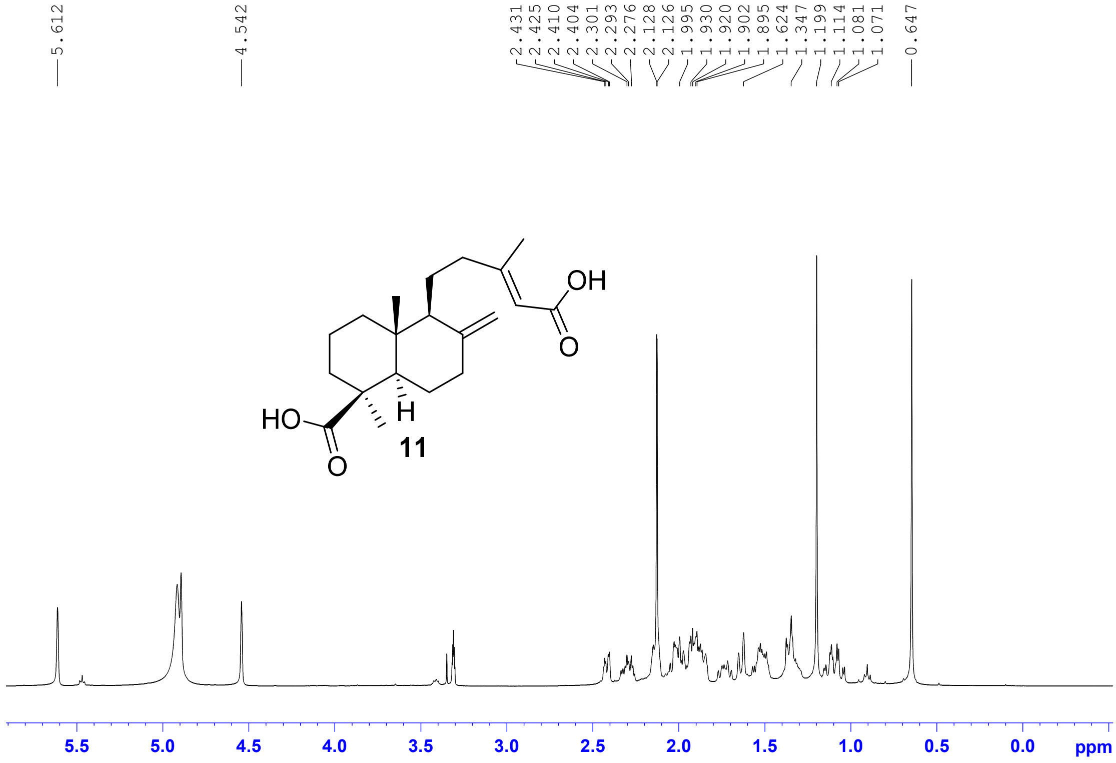

Figure S32 ^{1}H NMR Spectrum of **11** in Methanol-d_4

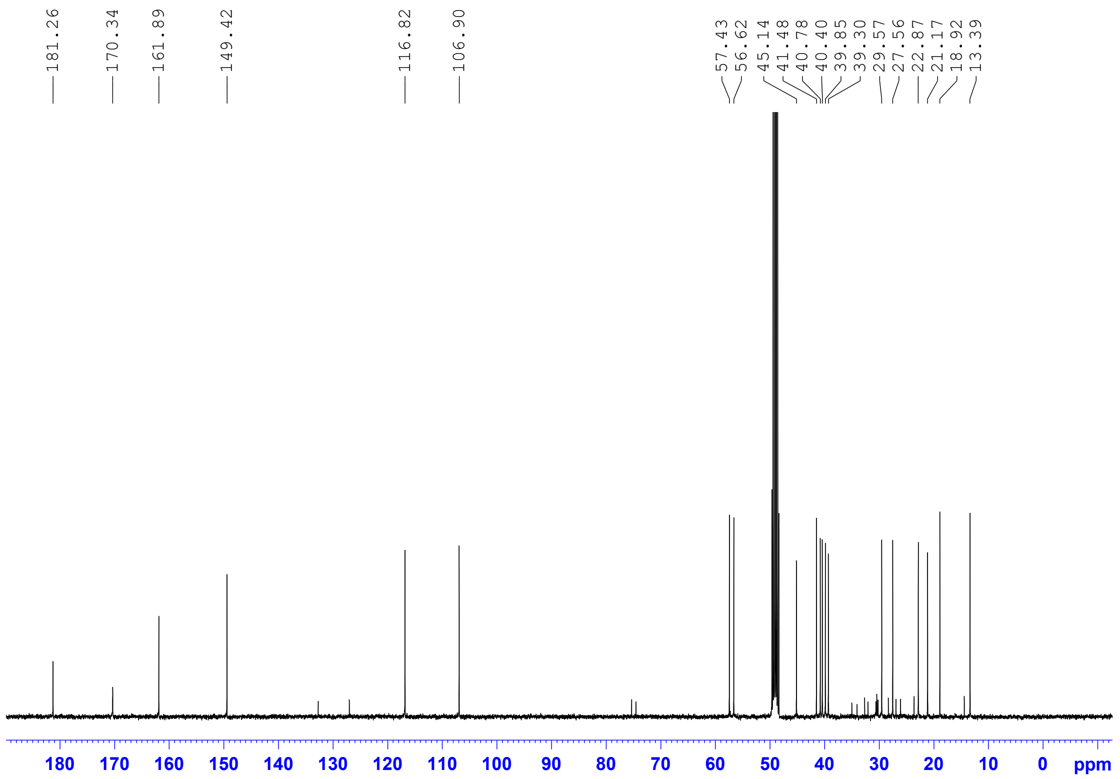

Figure S33 ^{13}C NMR Spectrum of **11** in Methanol-d_4

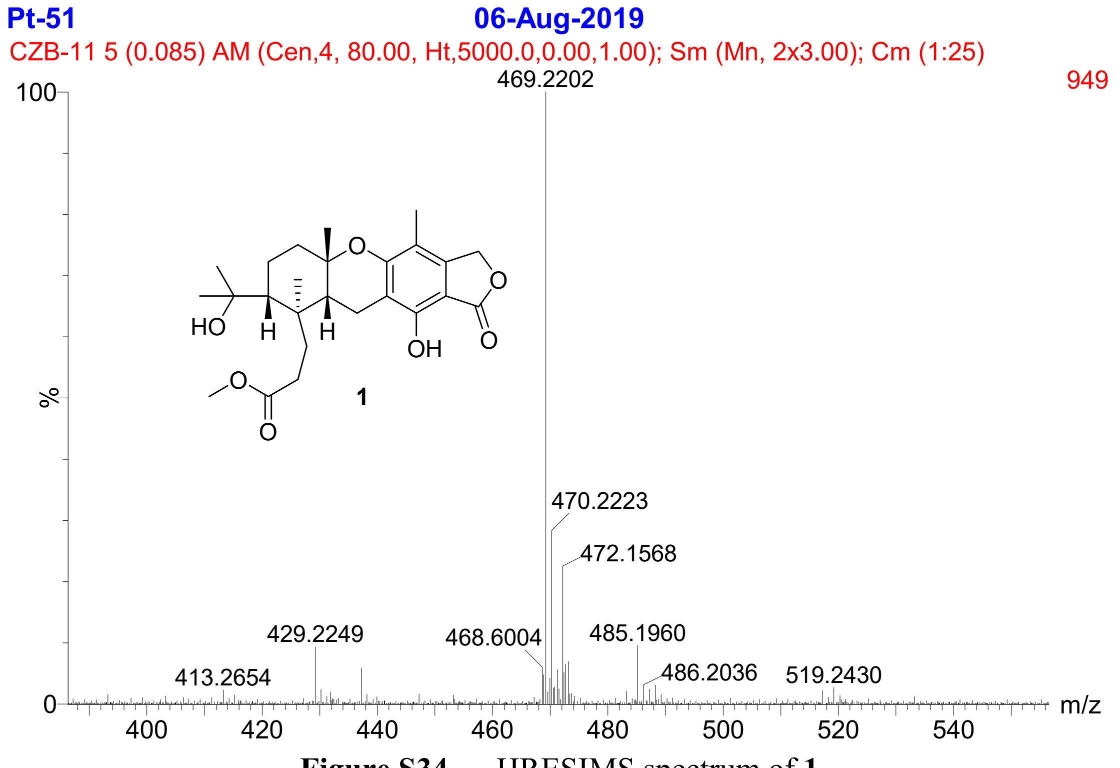

Figure S34 HRESIMS spectrum of **1**

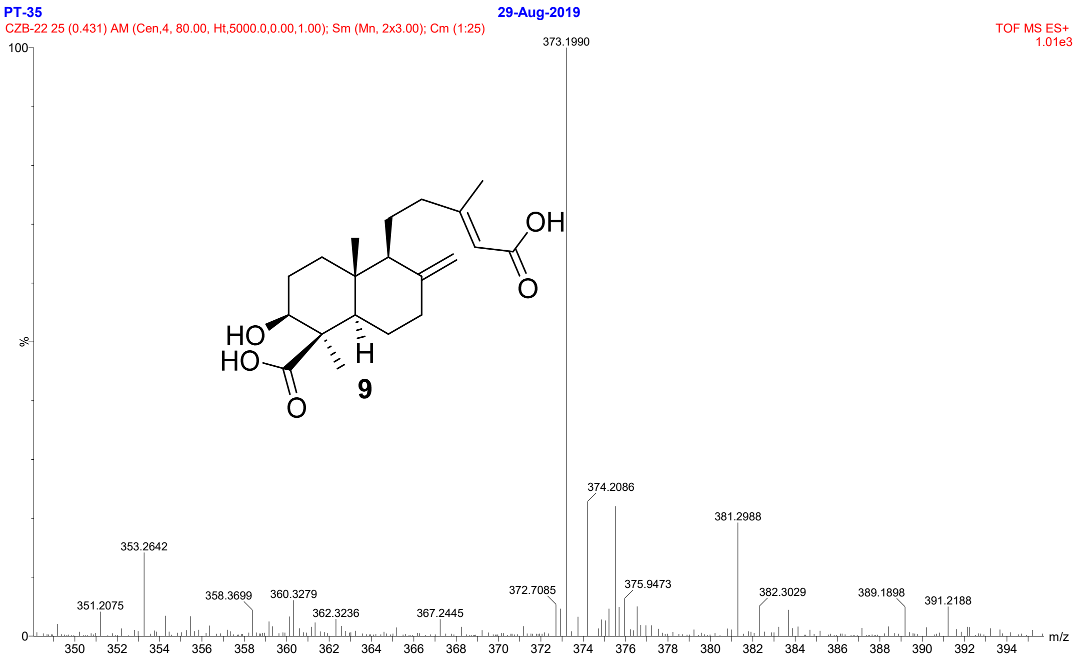

Figure S35 HRESIMS spectrum of **9**

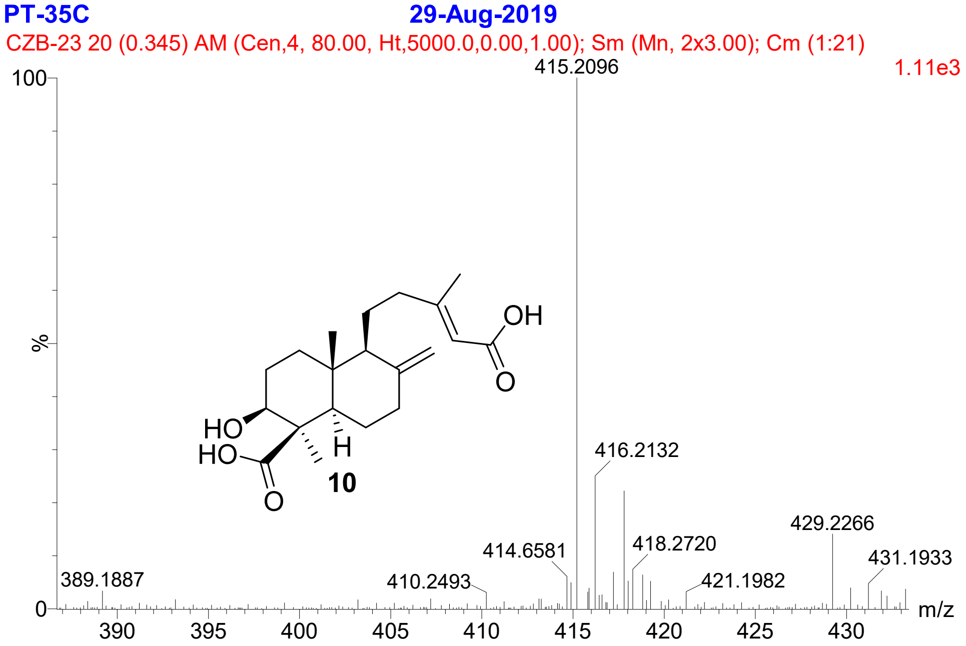

Figure S36 HRESIMS spectrum of **10**

S38 Details for ecd calculations of **1** and **9**

1. Computational Details.

In general, conformational analyses were carried out via random searching in the Sybyl-X 2.0 [1] using the MMFF94S force field with an energy cutoff of 3.0 kcal/mol. Subsequently, the conformers were re-optimized using density functional theory (DFT) at the b3lyp/6-31+g(d,p) level in MeOH using the polarizable conductor calculation model by the GAUSSIAN 09 program [2]. The energies, oscillator strengths, and rotational strengths (velocity) of the first 30 electronic excitations were calculated using the TDDFT methodology at the b3lyp/6-31+g(d,p) level in MeOH. The ECD spectra were simulated by the overlapping Gaussian function (half the bandwidth at 1/e peak height, $\sigma = 0.27$ for 11*S*, 14*R*, 20*S*, 21*R*-**1** and 0.3 for 3*S*, 4*R*, 5*R*, 9*S,* 10*R*-**9**) [3]. By comparing the experiment spectra with the calculated ECD spectra, the absolute configurations of **1**, **9**, and **10** were resolved.

1. Sybyl Software, version X 2.0; Tripos Associates Inc.: St. Louis, MO, 2013.

2. Frisch, M. J.; Trucks, G. W.; Schlegel, H. B.; Scuseria, G. E.; Robb, M. A.; Cheeseman, J. R.; Scalmani, G.; Barone, V.; Mennucci, B.; Petersson, G. A.; Nakatsuji, H.; Caricato, M.; Li, X.; Hratchian, H. P.; Izmaylov, A. F.; Bloino, J.; Zheng, G.; Sonnenberg, J. L.; Hada, M.; Ehara, M.; Toyota, K.; Fukuda, R.; Hasegawa, J.; Ishida, M.; Nakajima, T.; Honda, Y.; Kitao, O.; Nakai, H.; Vreven, T.; Montgomery, Jr., J. A.; Peralta, J. E.; Ogliaro, F.; Bearpark, M.; Heyd, J. J.; Brothers, E.; Kudin, K. N.; Staroverov, V. N.; Kobayashi, R.; Normand, J.; Raghavachari, K.; Rendell, A.; Burant, J. C.; Iyengar, S. S.; Tomasi, J.; Cossi, M.; Rega, N.; Millam, J. M.; Klene, M.; Knox, J. E.; Cross, J. B.; Bakken, V.; Adamo, C.; Jaramillo, J.; Gomperts, R.; Stratmann, R. E.; Yazyev, O.; Austin, A. J.; Cammi, R.; Pomelli, C.; Ochterski, J. W.; Martin, R. L.; Morokuma, K.; Zakrzewski, V. G.; Voth, G. A.; Salvador, P.; Dannenberg, J. J.; Dapprich, S.; Daniels, A. D.; Farkas, Ö.; Foresman, J. B.; Ortiz, J. V.; Cioslowski, J.; Fox, D. J.Gaussian 09, Rev. C 01; Gaussian, Inc., Wallingford CT, 2009.

3. Stephens, P. J.; Harada, N. ECD cotton effect approximated by the Gaussian curve and other methods. *Chirality* **2010**, *22*, 229–233.

Table S1. Energy analysis for 11*S*, 14*R*, 20*S*, 21*R*-1

conformer	Gibbs free energy (298.15 K)		
	G (Hartree)	ΔE (kcal/mol)	Population (%)
C1	-1499.8608818	0	40.89

C2	-1499.8604866	0.000395	26.89
C3	-1499.8599264	0.000955	14.85
C4	-1499.8593622	0.00152	8.17
C5	-1499.8593622	0.00152	8.17
C6	-1499.8574176	0.003464	1.04

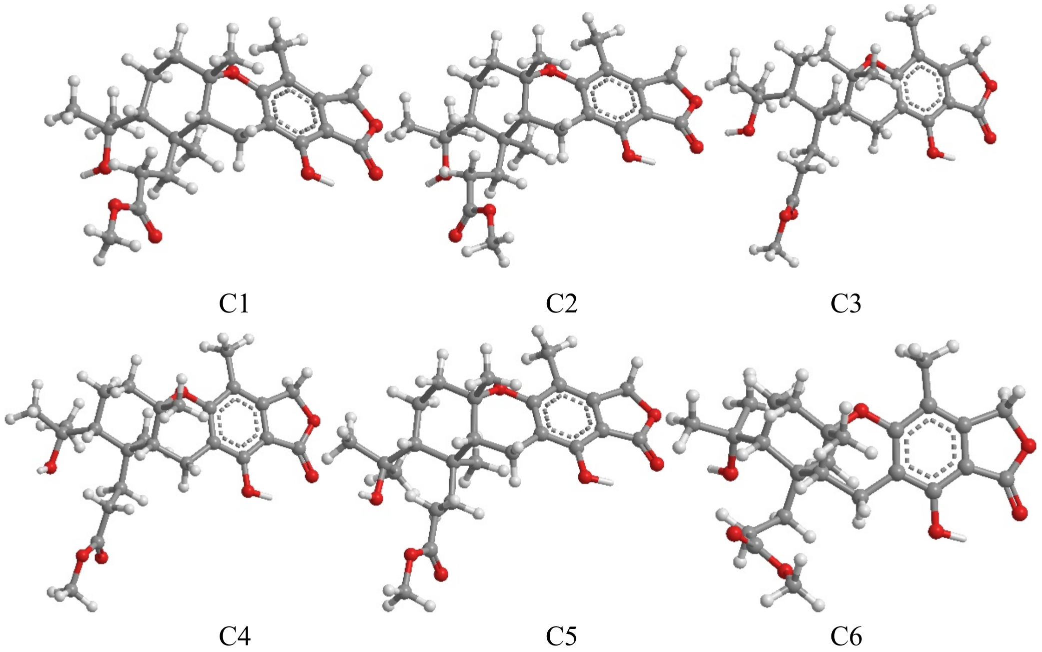

Figure S38. B3LYP-SCRF (PCM, methanol)/6-31G(d) optimized lowest energy conformers for 11*S*, 14*R*, 20*S*, 21*R*-1

Table S2. Calculated ECD Data for 11*S*, 14*R*, 20*S*, 21*R*-1

	C1		C2		C3	
State	Excitation energies(ev)	Rotatory Strengths*	Excitation energies(ev)	Rotatory Strengths*	Excitation energies(ev)	Rotatory Strengths*
1	4.3423	-10.1849	4.3432	-10.2701	4.3392	-9.5851
2	4.6544	-14.5714	4.6573	-13.4875	4.6508	-15.8288
3	5.3953	18.6966	5.3956	14.8889	5.3950	27.0698
4	5.4043	-40.6161	5.4046	-30.6921	5.4037	-50.9006
5	5.4244	31.1510	5.4231	28.0920	5.4265	33.1887
6	5.6561	4.9584	5.6564	3.2077	5.6594	4.6663
7	5.7113	1.4962	5.7189	-0.7876	5.6688	2.8995
8	5.7432	1.7120	5.7214	1.7861	5.7707	-0.2301
9	5.7977	-5.7033	5.7826	6.9826	5.8200	-2.9794
10	5.8477	-34.0495	5.7900	-18.5848	5.8502	-42.5731
11	5.8953	-8.5591	5.8461	-38.5442	5.9042	0.1414
12	5.9047	1.4478	5.9162	0.6205	5.9054	1.7291
13	5.9490	21.2693	5.9224	18.4753	5.9694	16.1350
14	5.9675	0.0957	5.9390	0.2110	6.0200	4.5258
15	5.9791	5.3116	5.9947	4.1796	6.0307	1.5716
16	6.0381	5.0929	6.0260	2.4399	6.0603	6.6554
17	6.0657	1.8406	6.0730	4.8799	6.0674	4.0735
18	6.1565	-5.2871	6.1612	-9.0049	6.1447	-5.9956

19	6.1972	-0.0130	6.1847	0.9770	6.1510	-0.2243
20	6.2190	4.9570	6.2064	8.1167	6.2250	4.0146
21	6.2659	-3.0170	6.2646	-0.6407	6.2524	-6.6939
22	6.2790	-0.3485	6.2968	-0.1736	6.2927	0.5157
23	6.4383	3.2705	6.4355	4.3680	6.4068	-4.7789
24	6.4503	0.3908	6.4405	-0.5025	6.4543	7.9211
25	6.4757	-2.8255	6.4627	-3.1545	6.4852	-2.4991
26	6.4892	-0.7179	6.5011	-2.7557	6.4994	-0.6109
27	6.5368	-26.7631	6.5343	-20.1978	6.5262	-17.3879
28	6.5442	6.8975	6.5499	0.6200	6.5583	-1.1859
29	6.5480	-0.3074	6.5675	0.6440	6.5637	-0.0416
30	6.5770	0.4094	6.5861	0.6410	6.5814	1.4085

State	C4		C5		C6	
	Excitation energies(ev)	Rotatory Strengths*	Excitation energies(ev)	Rotatory Strengths*	Excitation energies(ev)	Rotatory Strengths*
1	4.3394	-9.4963	4.3394	-9.4821	4.3418	-9.2010
2	4.6525	-15.4632	4.6526	-15.4487	4.6548	-11.2070
3	5.3918	27.8599	5.3919	27.8017	5.3939	11.1133
4	5.4025	-46.1109	5.4025	-46.0166	5.4038	-27.2933
5	5.4243	23.2449	5.4243	23.1910	5.4218	26.0566
6	5.5063	5.0155	5.5066	4.9951	5.5723	-0.2314
7	5.6553	5.5980	5.6553	5.5860	5.6527	2.5150
8	5.7621	0.1037	5.7621	0.1020	5.7004	15.0218
9	5.7757	-0.6904	5.7756	-0.7055	5.7085	0.6744
10	5.8459	-51.9862	5.8458	-51.8569	5.7614	-0.8375
11	5.8705	2.1775	5.8704	2.1688	5.7819	3.8384
12	5.8951	-3.4719	5.8951	-3.4442	5.8381	-59.8221
13	5.9290	30.1975	5.9293	30.0686	5.8755	37.1290
14	5.9712	-1.5763	5.9711	-1.5725	5.9307	2.4740
15	6.0406	2.4680	6.0406	2.4618	5.9850	5.7563
16	6.0634	11.4005	6.0633	11.4016	6.0139	1.1671
17	6.1148	-3.9200	6.1147	-3.9499	6.0674	3.7117
18	6.1731	0.0958	6.1734	0.0963	6.1415	0.6544
19	6.2329	3.6266	6.2328	3.6281	6.1661	-10.6797
20	6.2625	-2.0900	6.2624	-2.1071	6.2494	9.9309
21	6.2680	-11.4966	6.2680	-11.4825	6.2600	1.4891
22	6.3792	20.3547	6.3794	20.3296	6.3119	0.5731
23	6.4096	0.8348	6.4097	0.8304	6.4373	-8.4470
24	6.4222	-2.9996	6.4222	-3.0094	6.4452	11.8189
25	6.4410	-4.5657	6.4411	-4.4907	6.4662	-15.5996
26	6.4502	-2.2603	6.4505	-2.3350	6.5083	4.2941
27	6.4854	12.8157	6.4853	12.7508	6.5174	-5.4997
28	6.4914	3.6017	6.4914	3.6502	6.5433	2.6387
29	6.4975	3.7892	6.4974	3.7817	6.5635	-2.6095
30	6.5380	-1.1399	6.5380	-1.1394	6.5826	-0.5648

* R(velocity) 10**-40 erg-esu-cm

Table S3. Energy analysis for 3*S*, 4*R*, 5*R*, 9*S,* 10*R*-9

conformer	Gibbs free energy (298.15 K)		
	G (Hartree)	ΔE (kcal/mol)	Population (%)
C1	-1156.4235876	0.000000	56.71
C2	-1156.4232029	0.000385	37.72
C3	-1156.4213995	0.002188	5.57

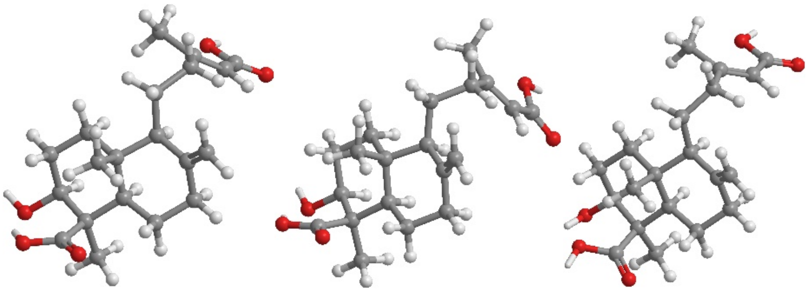

C1 C2 C3

Figure S39. B3LYP-SCRF (PCM, methanol)/6-31G(d) optimized lowest energy conformers for 3*S*, 4*R*, 5*R*, 9*S,* 10*R*-9

Table S4. Calculated ECD Data for 3*S*, 4*R*, 5*R*, 9*S,* 10*R*-9

State	C1		C2		C3	
	Excitation energies(ev)	Rotatory Strengths*	Excitation energies(ev)	Rotatory Strengths*	Excitation energies(ev)	Rotatory Strengths*
1	4.6010	0.1885	4.6085	1.0179	4.6150	0.2292
2	4.9383	-28.1188	4.9495	26.3398	4.9393	-28.3588
3	5.4430	28.8170	5.4568	-65.4959	5.4052	0.6885
4	5.5712	2.4772	5.5719	2.6024	5.4616	32.8551
5	5.6262	2.9246	5.6229	0.8594	5.5707	-3.0752
6	5.7118	0.8070	5.7088	0.4546	5.6432	2.8000
7	5.9114	7.6240	5.9135	4.6963	5.9272	-2.7408
8	5.9454	3.1454	5.9601	10.2782	5.9568	14.9799
9	6.1970	-2.6215	6.1922	-0.5705	5.9878	3.7343
10	6.2073	-22.3430	6.1980	-19.5587	6.0296	1.3518
11	6.2999	-3.3864	6.3013	-8.5403	6.1631	-1.9222
12	6.3025	-1.4133	6.3241	0.5372	6.1866	-29.4981
13	6.3424	0.8785	6.3435	-0.6169	6.3043	6.6005
14	6.4080	3.3274	6.4011	4.0959	6.3536	-1.9615
15	6.4418	2.4771	6.4086	-8.9424	6.3849	2.2176
16	6.5588	-8.0922	6.5734	4.6145	6.4169	13.9958
17	6.5663	-3.1322	6.5766	-14.0776	6.4441	-4.2821
18	6.6284	-0.1331	6.6421	0.2160	6.5218	1.1277
19	6.6585	-0.7568	6.6605	0.4400	6.5407	-13.8342
20	6.7071	-4.5083	6.6943	-6.6818	6.5723	1.0345
21	6.7468	18.9827	6.7328	-1.4261	6.6679	-1.2891
22	6.7492	-1.3379	6.7455	8.4490	6.6928	18.5719
23	6.7642	-2.1712	6.7574	7.2109	6.7063	-2.5368
24	6.7657	1.1022	6.7676	3.7896	6.7288	-0.7610
25	6.8090	9.7731	6.8120	0.8038	6.7586	1.2365

26	6.8196	2.4893	6.8218	-0.1712	6.8103	8.2468
27	6.8607	4.7353	6.8678	4.9920	6.8485	5.9794
28	6.8992	0.3276	6.9075	-2.6399	6.8779	-24.1421
29	6.9033	7.0166	6.9110	7.0137	6.8954	1.5147
30	6.9380	15.6876	6.9462	-5.4814	6.9200	12.7690

* R(velocity) 10**-40 erg-esu-cm

Table S1. Inhibitions (%) on NO production and cytotoxicity (%) toward RAW 264.7, MCF-7, and MDA-MB-468 cells (cell viability) of compounds 1−11.

no.	NO	RAW264.7	MCF7	MM468
**1**	97.80	33.15	104.04	31.61
**2**	56.83	46.16	117.22	78.25
**3**	-9.91	60.89	84.70	87.23
**4**	5.29	97.36	82.41	92.60
**5**	12.11	104.42	69.09	87.28
**6**	-3.74	74.58	73.83	92.29
**7**	-3.08	80.77	74.81	91.10
**8**	24.01	53.52	76.26	79.27
**9**	7.81	83.30	88.78	92.14
**10**	72.25	58.40	89.96	94.51
**11**	4.85	89.86	83.52	89.27
